# Supplementary material for: Preparation of Modified Chitosan Microsphere-Supported Copper Catalysts for the Borylation of α,β-Unsaturated Compounds
Source: Polymers (Basel). 2019 Aug 28;11(9):1417. doi: 10.3390/polym11091417 (PMC6788198; doi:10.3390/polym11091417)

Supplementary information for

Preparation of Modified Chitosan Microspheres Supported Copper Catalysts for The Borylation of α,β-Unsaturated Compounds

Wei Wang[1], Zufeng Xiao[1], Chaofan Huang[1], Kewang Zheng[1], Yin Luo[1], Yumin Dong[1], Zitong Shen[1], Wei Li[1,*], Caiqin Qin[1,2 **]

[1] School of Chemistry and Materials Science, Hubei Engineering University, Xiaogan 432000, China

[2] Key Laboratory of Biological Resources and Environmental Biotechnology, Hubei Province

Wei Wang[1] and Zufeng Xiao[1] are contributed equally.

*Correspondence to: Wei Li (E-mail: weili@hbeu.edu.cn)

** Correspondence to: Caiqin Qin (E-mail: *qincq*@hbeu.edu.cn)

General Procedure for CSM@Cu^{0}Catalyzed Borylation of α,β-Unsaturated Acceptors in Aqueous Media

In a sample vial, acetone (0.5 mL) and H_2O (0.5 mL) were successively added to a mixture of CSM@Cu^0 (3.8 mg, 1 mol %) and $B_2(pin)_2$ (60.9 mg, 0.24 mmol) under air. The reaction mixture was stirred for 0.5 h at room temperature, followed by successive addition of substrate (**4**) (0.2 mmol). After stirring for 8h at room temperature, the reaction mixture was filtered and the filtrate was extracted with EtOAc (20 mL) three times. The combined organic phase was concentrated under reduced pressure and the residue was dissolved in THF (1 mL) and H_2O (0.5 mL). Excess amount of $NaBO_3$•$4H_2O$ (244 mg) was then added and the mixture was stirred at room temperature for 4 h. The aqueous layer was extracted with EtOAc (20 mL) three times, and the combined organic layers were dried over anhydrous Na_2SO_4. After concentrated under reduced pressure, the crude mixture was purified by preparative TLC to afford the desired product **6**.

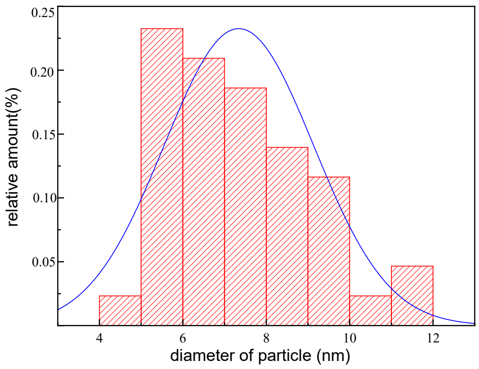

Figure S1. Diameter distribution of copper nanoparticles (CSM@Cu^0)

Table 1S. The content of N, C, H, O of chitosan microspheres (CS) and of modified chitosan microspheres (CSM)

Compound	H(w%)	C(w%)	N(w%)	O(w%)	C:N:O(Atomic%:Atomic%:Atomic%)
CS	6.81	44.91	8.63	39.65	6.04:0.994:4
CSM	5.93	54.23	9.72	30.12	9.6: 1.473:4

Table 2S. The adsorption of the CS and CSM

Entry	Conc. before adsorption (C_0, mg/L)	Adsorption Time (min)	Conc. after adsorption (Ci, mg/L)	Adsorption conc. (mg/L)	Adsorption (%)	Absorption capacity (qi, mg/g)
1[a]	100.63	180	2.62	98.01	97.40	98.01
2[b]	100.32	180	42.71	57.61	57.43	57.43

a. CSM, pH =5; b. CS, pH =5

Table. 3S Swelling capability of CSM at different pH value

Entry	pH	Initial quality (m_0, g)	Quality after swelling for 3h (m_i, g)	Quality after drying (m_f, g)	Swelling capability (SC= $\frac{mi-mf}{mf} \times 100\%$)
1	2	0.1036	0.2766	0.0812	240.6
2	3	0.1070	0.2480	0.0939	164.1
3	4	0.1047	0.1528	0.0965	58.3
4	5	0.0999	0.1465	0.0977	49.9

All products are literature-known; obtained analytical data is in full agreement with reported data.

6a. 3-Hydroxy-1,3-diphenylpropan-1-one

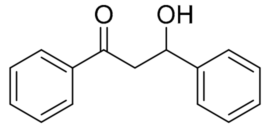

Colorless oil

^{1}H NMR (400 MHz, Chloroform-d) δ 7.96 (d, J = 8.0 Hz, 2H), 7.62 – 7.55 (m, 1H), 7.46 (dd, J = 11.1, 7.6 Hz, 4H), 7.39 (t, J = 7.5 Hz, 2H), 7.34 – 7.28 (m, 1H), 5.36 (t, J = 6.2 Hz, 1H), 3.61 (br, 1H), 3.41 – 3.33 (m, 2H).

^{13}C NMR (100 MHz, Chloroform-d) δ = 199.9, 143.0, 136.5, 133.4, 128.5, 128.4, 128.0, 127.5, 125.6, 69.9, 47.3.

6b. 3-Hydroxy-3-phenyl-1-(*p*-tolyl)propan-1-one

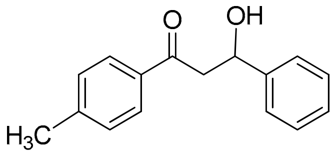

White solid

^{1}H NMR (400 MHz, Chloroform-d) δ 7.86 (d, J = 8.3 Hz, 2H), 7.46 – 7.42 (m, 2H), 7.41 – 7.36 (m, 2H), 7.31 (d, J = 7.3 Hz, 1H), 7.29 – 7.24 (m, 4H), 5.34 (t, J = 7.5 Hz, 1H), 3.66 (br, 1H), 3.38 – 3.31 (m, 2H), 2.42 (s, 3H).

^{13}C NMR (101 MHz, Chloroform-d) δ 199.8, 144.6, 143.0, 134.1, 129.3, 128.5, 128.2, 127.6, 125.72, 70.1, 47.2, 21.7.

6c. 3-Hydroxy-3-phenyl-1-(*m*-tolyl)propan-1-one

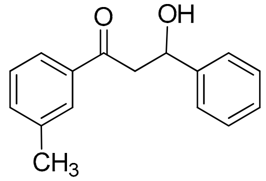

White solid

^{1}H NMR (400 MHz, Chloroform-d) δ 7.78 – 7.73 (m, 2H), 7.47 – 7.43 (m, 2H), 7.42 – 7.28 (m, 5H), 5.34 (td, J = 6.1, 2.0 Hz, 1H), 3.70 (s, 1H), 3.37 (d, J = 6.0 Hz, 2H), 2.41 (s, 3H).
^{13}C NMR (101 MHz, Chloroform-d) δ 200.3, 142.9, 138.4, 136.5, 134.3, 128.6, 128.5, 128.5, 127.6, 125.7, 125.3, 69.9, 47.3, 21.3.
4. 3-Hydroxy-1-(4-methoxyphenyl)-3-phenylpropan-1-one

6d 3-hydroxy-1-(4-methoxyphenyl)-3-phenylpropan-1-one

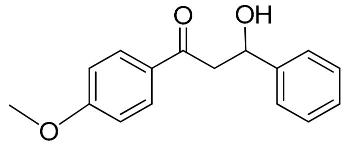

White solid

^{1}H NMR (400 MHz, Chloroform-d) δ 7.96 – 7.90 (m, 2H), 7.47 – 7.42 (m, 2H), 7.41 – 7.34 (m, 2H), 7.31 (d, J = 7.2 Hz, 1H), 6.93 (d, J = 8.9 Hz, 2H), 5.37 – 5.29 (m, 1H), 3.87 (s, 3H), 3.78 –3.73 (br, 1H), 3.37 – 3.24 (m, 2H).
^{13}C NMR (101 MHz, Chloroform-d) δ 199.8, 164.0， 144.6, 143.0, 134.1, 129.3, 128.50 128.24 127.6, 125.7, 70.1, 47.2.

6e. 1-(4-fluorophenyl)-3-hydroxy-3-phenylpropan-1-one

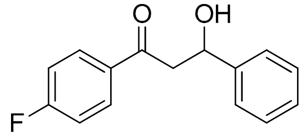

Colorless oil

^{1}H NMR (400 MHz, Chloroform-d) δ 8.02 – 7.93 (m, 2H), 7.47 – 7.41 (m, 2H), 7.41 – 7.35 (m, 2H), 7.34–7.27 (m, 1H), 7.18 – 7.08 (m, 2H), 5.40 – 5.25 (m, 1H), 3.48 (d, J = 2.9 Hz, 1H), 3.42 –3.26 (m, 2H).
^{13}C NMR (101 MHz, Chloroform-d) δ 198.4, 167.2, 164.7, 142.8, 133.0, 133.0, 130.8, 130.8, 128.5, 127.7, 125.7, 115.9, 115.7, 70.0, 47.3.

6f. 3-Hydroxy-1-phenyl-3-(*p*-tolyl)propan-1-one

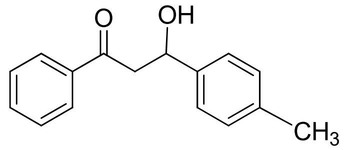

Colorless oil

^{1}H NMR (400 MHz, Chloroform-d) δ 7.99 – 7.92 (m, 2H), 7.61 – 7.55 (m, 1H), 7.51 – 7.42 (m, 2H), 7.36 – 7.30 (m, 2H), 7.19 (d, J = 7.8 Hz, 2H), 5.32 (s, 1H), 3.54 (d, J = 2.7 Hz, 1H), 3.45 – 3.30 (m, 2H), 2.36 (s, 3H).
^{13}C NMR (101 MHz, Chloroform-d) δ 200.0, 140.0, 137.2, 136.5, 133.5, 129.1, 128.6, 128.0, 125.6, 69.8, 47.34, 21.06.

6g. 3-Hydroxy-3-(4-methoxyphenyl)-1-phenylpropan-1-one

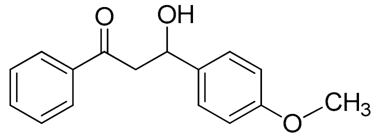

White solid

^{1}H NMR (400 MHz, Chloroform-d) δ 7.99 – 7.93 (m, 2H), 7.61 – 7.55 (m, 1H), 7.47 (dd, J = 8.4, 7.0 Hz, 2H), 7.41 – 7.34 (m, 2H), 6.95 – 6.88 (m, 2H), 5.30 (s, 1H), 3.81 (s, 3H), 3.52 (d, J = 2.8 Hz, 1H), 3.43 – 3.29 (m, 2H).
^{13}C NMR (101 MHz, Chloroform-d) δ 200.0, 158.9, 136.5, 135.1, 133.4, 128.5, 128.0, 126.9, 113.8, 69.6, 55.1, 47.2.

6h. 3-(4-fluorophenyl)-3-hydroxy-1-phenylpropan-1-one

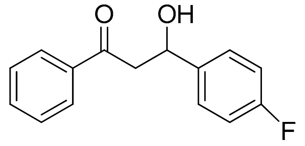

Colorless oil

^{1}H NMR (400 MHz, Chloroform-d) δ 8.00 – 7.92 (m, 2H), 7.64 – 7.56 (m, 1H), 7.47 (t, J = 7.7 Hz, 2H), 7.44 – 7.38 (m, 2H), 7.12 – 7.01 (m, 2H), 5.52 – 5.08 (m, 1H), 3.62 (d, J = 2.9 Hz, 1H), 3.49 – 3.07 (m, 2H).
^{13}C NMR (101 MHz, Chloroform-d) δ 200.00, 163.4, 160.9, 138.7, 138.7, 136.5, 133.7, 128.7, 128.1, 127.4, 127.3, 115.4, 115.2, 69.4, 47.3.

6i. 3-(4-chlorophenyl)-3-hydroxy-1-phenylpropan-1-one

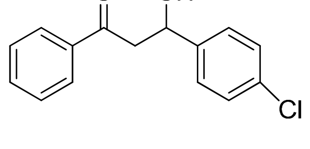

Colorless oil

^1H NMR (400 MHz, Chloroform-d) δ 7.98 – 7.91 (m, 2H), 7.64 – 7.56 (m, 1H), 7.50 – 7.43 (m, 2H), 7.40 – 7.31 (m, 4H), 5.33 (dt, J = 7.6, 3.7 Hz, 1H), 3.64 (q, J = 3.0 Hz, 1H), 3.40 – 3.25 (m, 2H).
^{13}C NMR (101 MHz, Chloroform-d) δ 199.8, 141.4, 136.3, 133.7, 133.2, 128.6, 128.6, 128.0, 127.1, 127.1, 77.3, 76.7, 69.3, 47.2.

6j. 3-(4-bromophenyl)-3-hydroxy-1-phenylpropan-1-one

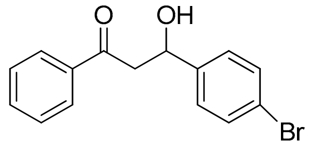

Colorless oil

^1H NMR (400 MHz, Chloroform-d) δ 7.97 – 7.92 (m, 2H), 7.64 – 7.57 (m, 1H), 7.54 – 7.44 (m, 4H), 7.34 (s, 2H), 5.30 (s, 2H), 3.63 (d, J = 3.0 Hz, 1H), 3.41 – 3.24 (m, 2H).
^{13}C NMR (101 MHz, Chloroform-d) δ 199.9, 141.9, 136.4, 133.8, 131.6, 128.7, 128.1, 127.5, 121.4, 69.4, 47.2.

6k. 3-(3-bromophenyl)-3-hydroxy-1-phenylpropan-1-one

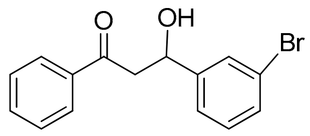

Colorless oil

^1H NMR (400 MHz, Chloroform-d) δ 8.02 – 7.96 (m, 2H), 7.67 – 7.61 (m, 2H), 7.55 – 7.44 (m, 3H), 7.39 (dt, J = 7.9, 1.4 Hz, 1H), 7.31 – 7.27 (m, 1H), 5.36 (d, J = 4.6 Hz, 1H), 3.79 (d, J = 3.1 Hz, 1H), 3.56 – 3.15 (m, 2H).
^{13}C NMR (101 MHz, Chloroform-d) δ 199.8, 145.2, 136.2, 133.7, 130.6, 130.1, 128.8, 128.7, 128.1, 124.3, 122.6, 69.2, 47.1.

6l 3-Hydroxy-1-phenyl-3-(4-(trifluoromethyl)phenyl)propan-1-one

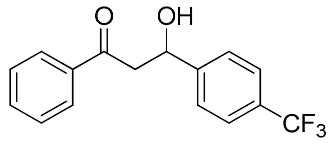

Colorless oil

^1H NMR (400 MHz, Chloroform-d) δ 7.99 – 7.92 (m, 2H), 7.64 (d, J = 8.2 Hz, 2H), 7.58 (dd, J = 11.8, 7.8 Hz, 3H), 7.48 (t, J = 7.8 Hz, 2H), 7.37 (d, J = 5.8 Hz, 1H), 5.45 – 5.37 (m, 1H), 3.72 (d, J = 3.1 Hz, 1H), 3.46 – 3.29 (m, 3H).

^{13}C NMR (101 MHz, Chloroform-d) δ 199.8, 146.9, 136.3, 133.8, 128.8, 128.1, 126.0, 125.5, 125.5, 69.4, 47.2.

6m. 3-(4-fluorophenyl)-3-hydroxy-1-(p-tolyl)propan-1-one

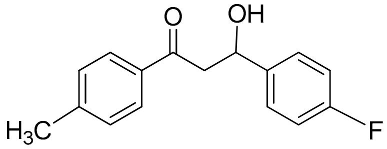

Colorless oil

^{1}H NMR (400 MHz, Chloroform-d) δ 7.89 – 7.81 (m, 2H), 7.45 – 7.36 (m, 2H), 7.30 – 7.23 (m, 3H), 7.11 – 7.01 (m, 2H), 5.37 – 5.27 (m, 1H), 3.71 (d, J = 2.8 Hz, 1H), 3.41 – 3.22 (m, 2H), 2.42 (s, 3H).
^{13}C NMR (101 MHz, Chloroform-d) δ 199.6, 163.3, 160.8, 144.6, 138.8, 138.7, 133.9, 129.3, 128.2, 127.4, 127.3, 115.3, 115.1, 77.3, 76.9, 76.6, 69.4, 47.1, 21.6.

6n. 3-Hydroxy-1,3-bis(4-methoxyphenyl)propan-1-one

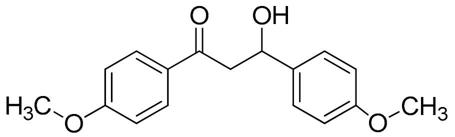

White solid

^{1}H NMR (400 MHz, Chloroform-d) δ 7.97 – 7.88 (m, 2H), 7.42 – 7.32 (m, 2H), 6.91 (t, J = 8.4 Hz, 4H), 5.26 (td, J = 6.2, 2.5 Hz, 1H), 3.83 (d, J = 23.1 Hz, 6H), 3.72 (t, J = 2.4 Hz, 1H), 3.29 (d, J = 6.1 Hz, 2H).
^{13}C NMR (101 MHz, Chloroform-d) δ 198.8, 163.9, 159.1, 135.3, 130.5, 129.7, 127.0, 113.9, 113.8, 69.8, 55.5, 55.3, 46.9.

6o. 3-(2-chlorophenyl)-3-hydroxy-1-(4-methoxyphenyl)propan-1-one

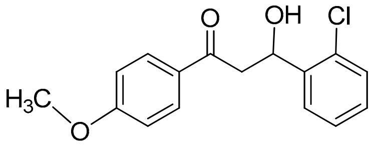

White solid

^{1}H NMR (400 MHz, Chloroform-d) δ 7.97 – 7.90 (m, 2H), 7.72 (dd, J = 7.6, 1.7 Hz, 1H), 7.37 – 7.30 (m, 2H), 7.23 (td, J = 7.6, 1.7 Hz, 1H), 6.95 – 6.89 (m, 2H), 5.66 (d, J = 9.7 Hz, 1H), 4.06 (dt, J = 3.1, 1.5 Hz, 1H), 3.86 (d, J = 0.9 Hz, 3H), 3.58 – 3.44 (m, 1H), 3.07 (dd, J = 17.5, 9.6 Hz, 1H).
^{13}C NMR (101 MHz, Chloroform-d) δ 198.8, 163.9, 140.4, 131.0, 130.5, 129.4, 129.2, 128.4, 127.2, 127.2, 113.8, 66.9, 55.7, 44.7.

6p. 3-(4-bromophenyl)-3-hydroxy-1-(4-methoxyphenyl)propan-1-one

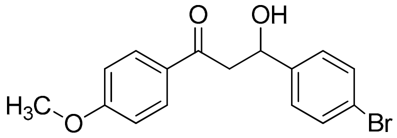

White solid

^{1}H NMR (400 MHz, Chloroform-d) δ 7.92 (d, J = 9.0 Hz, 2H), 7.50 (d, J = 8.5 Hz, 2H), 7.32 (d, J = 8.2 Hz, 2H), 6.94 (d, J = 9.0 Hz, 2H), 5.32 – 5.25 (m, 1H), 3.88 (s, 3H), 3.80 (d, J = 2.9 Hz, 1H), 3.37 – 3.18 (m, 2H).
^{13}C NMR (101 MHz, Chloroform-d) δ 198.4, 163.9, 142.1, 131.5, 130.5, 129.4, 127.4, 121.2, 113.8, 69.5, 55.4, 46.6.

6q 1-(4-fluorophenyl)-3-hydroxy-3-(4-methoxyphenyl)propan-1-one

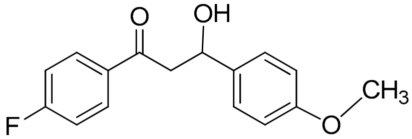

White solid

^{1}H NMR (400 MHz, Chloroform-d) δ 8.01 – 7.93 (m, 2H), 7.39 – 7.32 (m, 2H), 7.17 – 7.08 (m, 2H), 6.94 – 6.86 (m, 2H), 5.28 (d, J = 8.8 Hz, 1H), 3.81 (s, 3H), 3.51 – 3.44 (m, 1H), 3.40 – 3.22 (m, 2H).
^{13}C NMR (101 MHz, Chloroform-d) δ 198.5, 167.3, 164.7, 159.1, 135.0, 133.1, 133.0, 130.9, 130.8, 127.0, 115.9, 115.7, 113.9, 69.6, 55.3, 47.3, 29.7.

6r. 3-(4-chlorophenyl)-1-(4-fluorophenyl)-3-hydroxypropan-1-one

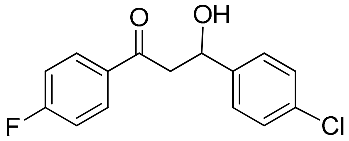

Colorless oil

^{1}H NMR (400 MHz, Chloroform-d) δ 8.02 – 7.92 (m, 2H), 7.35 (dd, J = 3.2, 1.6 Hz, 4H), 7.14 (t, J = 8.4 Hz, 2H), 5.31 (t, J = 6.1 Hz, 1H), 3.60 (d, J = 6.4 Hz, 1H), 3.36 – 3.23 (m, 2H).
^{13}C NMR (101 MHz, Chloroform-d) δ 198.2, 167.3, 164.8, 141.2, 133.3, 132.8, 132.8, 130.8, 130.7, 128.7, 127.1, 116.0, 115.8, 69.3, 47.1.

6s. 3-(4-bromophenyl)-1-(4-fluorophenyl)-3-hydroxypropan-1-one

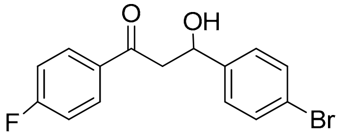

Colorless oil

^{1}H NMR (400 MHz, Chloroform-d) δ 7.98 (ddd, J = 8.8, 5.3, 1.4 Hz, 2H), 7.51 (dd, J = 8.3, 1.4 Hz, 2H), 7.32 (dd, J = 8.3, 1.3 Hz, 2H), 7.14 (td, J = 8.6, 1.4 Hz, 2H), 5.31 (d, J = 3.2 Hz, 1H), 3.55 (dd, J = 3.0, 1.2 Hz, 1H), 3.31 (dd, J = 5.8, 1.1 Hz, 2H).
^{13}C NMR (101 MHz, Chloroform-d) δ 197.9, 167.2, 164.7, 141.9, 132.8, 132.8, 131.5, 130.8, 130.7, 127.4, 121.3, 115.9, 115.7, 69.2, 47.1.

6t. 4-Hydroxy-4-phenylbutan-2-one

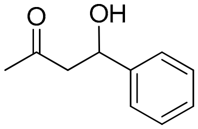

Colorless oil

^{1}H NMR (400 MHz, Chloroform-d) δ 7.29 – 7.24 (m, 4H), 7.22 – 7.17 (m, 1H), 5.06 (dd, J = 9.1, 3.4 Hz, 1H), 3.26 (s, 1H), 2.88 – 2.65 (m, 2H), 2.10 (s, 3H).
^{13}C NMR (101 MHz, Chloroform-d) δ 208.9, 142.7, 128.4, 127.6, 125.5, 69.8, 51.9, 30.7.

6u. 1-Hydroxy-4,4-dimethyl-1-phenylpentan-3-one

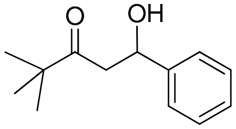

Colorless oil

^{1}H NMR (400 MHz, Chloroform-d) δ 7.29 – 7.21 (m, 4H), 7.20 – 7.14 (m, 1H), 5.03 (d, J = 3.0 Hz, 1H), 3.47 (d, J = 3.0 Hz, 1H), 2.81 – 2.73 (m, 2H), 1.03 (s, 9H).
^{13}C NMR (101 MHz, Chloroform-d) δ 216.7, 143.0, 128.4, 127.4, 125.6, 70.0, 45.4, 44.3, 26.1.

6v. 3-Hydroxy-3-(naphthalen-1-yl)-1-phenylpropan-1-one

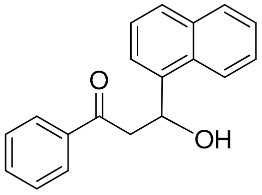

White solid

^{1}H NMR (400 MHz, Chloroform-d) δ 8.09 – 8.03 (m, 1H), 7.99 – 7.93 (m, 2H), 7.92 – 7.87 (m, 1H), 7.84 – 7.78 (m, 2H), 7.63 – 7.41 (m, 6H), 6.16 (dt, J = 9.3, 2.5 Hz, 1H), 3.76 (d, J = 2.8 Hz, 1H), 3.62 – 3.38 (m, 2H).

^{13}C NMR (101 MHz, Chloroform-d) δ 200.3, 138.4, 136.4, 133.7, 133.6, 129.8, 129.0, 128.6, 128.1, 128.0, 126.2, 125.6, 125.5, 123.1, 122.7, 66.7, 46.7.

6w. 3-hydroxy-1-phenyl-3-(thiophen-2-yl)propan-1-one

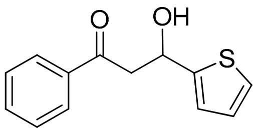

Colorless oil

^{1}H NMR (400 MHz, Chloroform-d) δ 8.00 – 7.91 (m, 2H), 7.62 – 7.53 (m, 1H), 7.46 (t, J = 7.6 Hz, 2H), 7.28 – 7.21 (m, 1H), 7.02 (dd, J = 3.5, 1.1 Hz, 1H), 6.97 (dd, J = 4.9, 3.6 Hz, 1H), 5.64 – 5.52 (m, 1H), 3.77 – 3.69 (m, 1H), 3.57 – 3.41 (m, 2H).
^{13}C NMR (101 MHz, Chloroform-d) δ 199.4, 146.6, 136.3, 133.6, 128.6, 128.0, 126.6, 124.6, 123.5, 66.4, 47.1.

6x 3-Hydroxy-3-(thiophen-2-yl)-1-(m-tolyl)propan-1-one

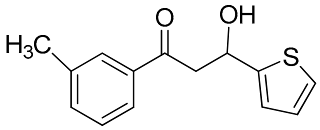

Colorless oil

^{1}H NMR (400 MHz, Chloroform-d) δ 7.79 – 7.73 (m, 2H), 7.44 – 7.33 (m, 2H), 7.30 – 7.25 (m, 1H), 7.06 – 7.02 (m, 1H), 6.99 (dd, J = 5.0, 3.5 Hz, 1H), 5.60 (s, 1H), 3.81 (s, 1H), 3.57 – 3.43 (m, 2H), 2.42 (s, 3H).
^{13}C NMR (101 MHz, Chloroform-d) δ 199.8, 146.6, 138.5, 136.4, 134.5, 128.6, 128.6, 126.7, 125.3, 124.6, 123.5, 66.4, 47.1, 21.3.

6y. 1-(4-fluorophenyl)-3-hydroxy-3-(thiophen-2-yl)propan-1-one

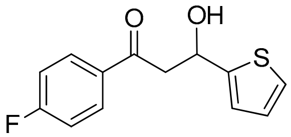

Colorless oil

^{1}H NMR (400 MHz, Chloroform-d) δ 8.00 (dd, J = 8.7, 5.5 Hz, 2H), 7.30 – 7.25 (m, 1H), 7.15 (t, J = 8.6 Hz, 2H), 7.04 (d, J = 3.4 Hz, 1H), 6.99 (dd, J = 5.1, 3.6 Hz, 1H), 5.60 (d, J = 7.2 Hz, 1H), 3.64 (d, J = 3.1 Hz, 1H), 3.56 – 3.39 (m, 2H), 1.26 (s, 1H).
^{13}C NMR (101 MHz, Chloroform-d) δ 197.8, 167.3, 164.8, 146.5, 132.9, 130.9, 130.8, 126.7, 124.7, 123.5, 116.0, 115.7, 66.5, 47.1, 29.7.

6z 3-Hydroxy-1,5-diphenylpent-4-en-1-one

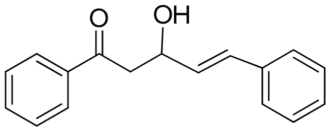

Colorless oil

^{1}H NMR (400 MHz, Chloroform-*d*) δ 8.01 – 7.95 (m, 2H), 7.59 (d, J = 7.4 Hz, 1H), 7.49 (dd, J = 8.4, 7.1 Hz, 2H), 7.42 – 7.36 (m, 2H), 7.36 – 7.29 (m, 2H), 7.28 – 7.20 (m, 2H), 6.72 (dd, J = 16.0, 1.3 Hz, 1H), 6.32 (dd, J = 15.9, 6.1 Hz, 1H), 4.96 (d, J = 6.3 Hz, 1H), 3.43 – 3.38 (m, 1H), 3.32 – 3.27 (m, 2H).
^{13}C NMR (101 MHz, Chloroform-d) δ 200.0, 136.5, 136.5, 133.6, 130.4, 130.2, 128.7, 128.6, 128.1, 127.7, 126.4, 68.6, 45.1, 29.6.

6a

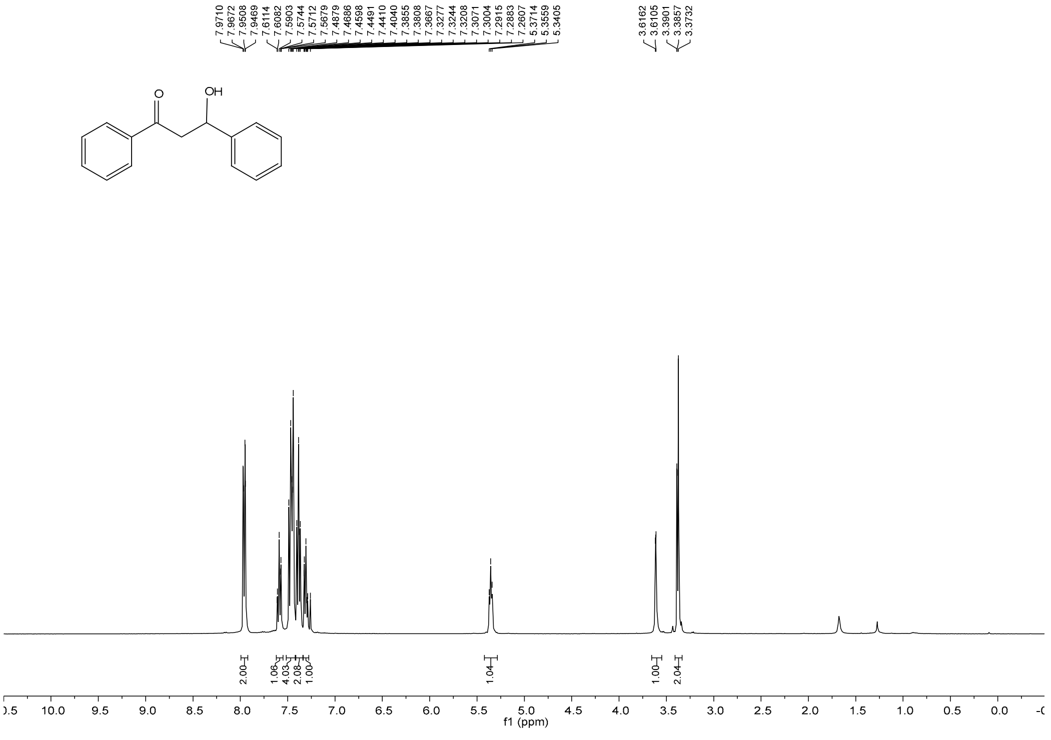

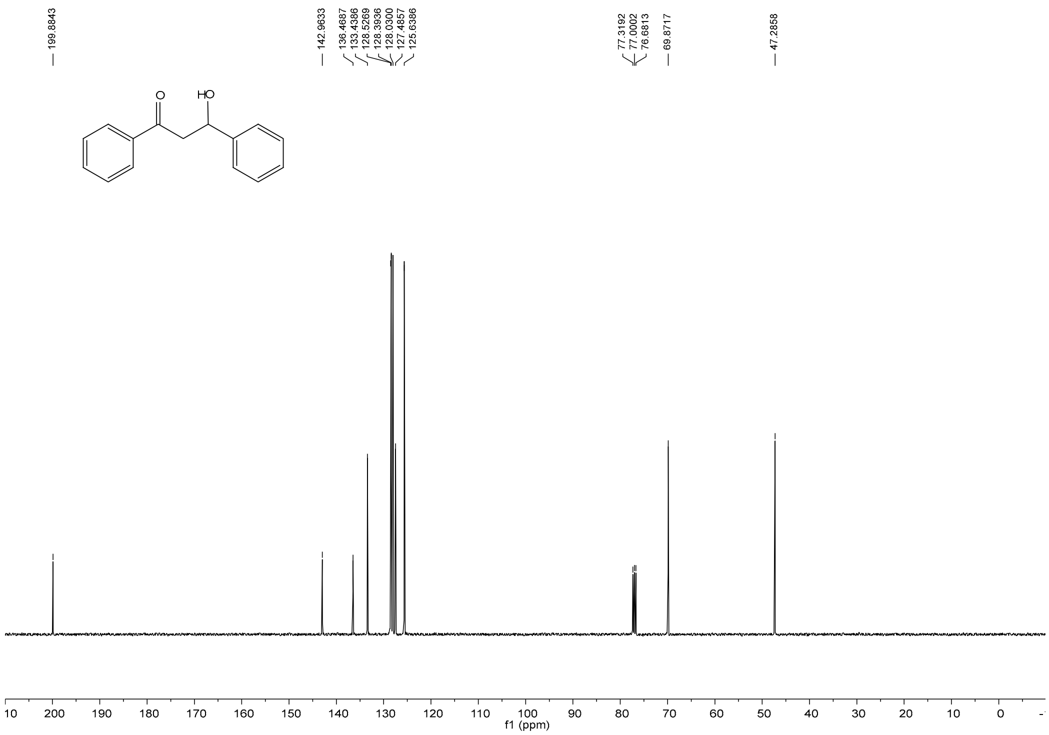
199.8843
142.9633
136.4687
133.4386
128.5269
128.3936
128.0300
127.4857
125.6386
77.3192
77.0002
76.6813
69.8717
47.2858
O
HO
f1 (ppm)

6b

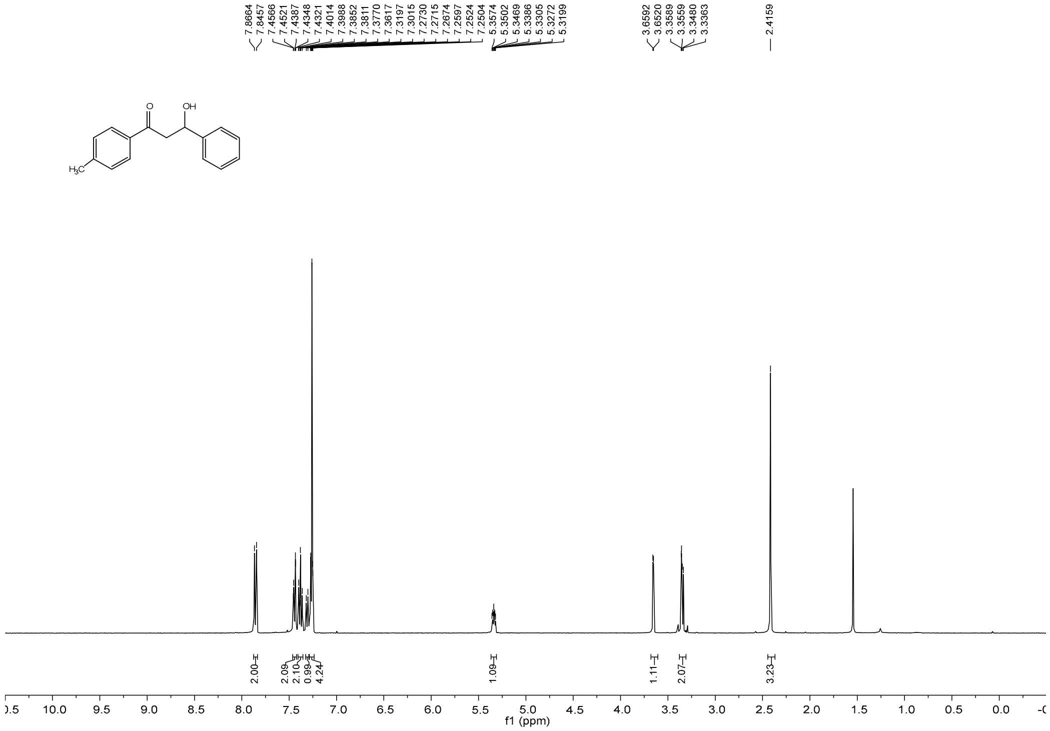
7.8664
7.8457
7.4566
7.4521
7.4387
7.4348
7.4321
7.4014
7.3988
7.3852
7.3811
7.3770
7.3617
7.3197
7.3015
7.2730
7.2715
7.2674
7.2597
7.2524
7.2504
5.3574
5.3502
5.3469
5.3386
5.3305
5.3272
5.3199
3.6592
3.6520
3.3589
3.3559
3.3480
3.3363
2.4159
O
OH
H_3C
2.00
2.09
2.10
0.99
4.24
1.09
1.11
2.07
3.23
f1 (ppm)

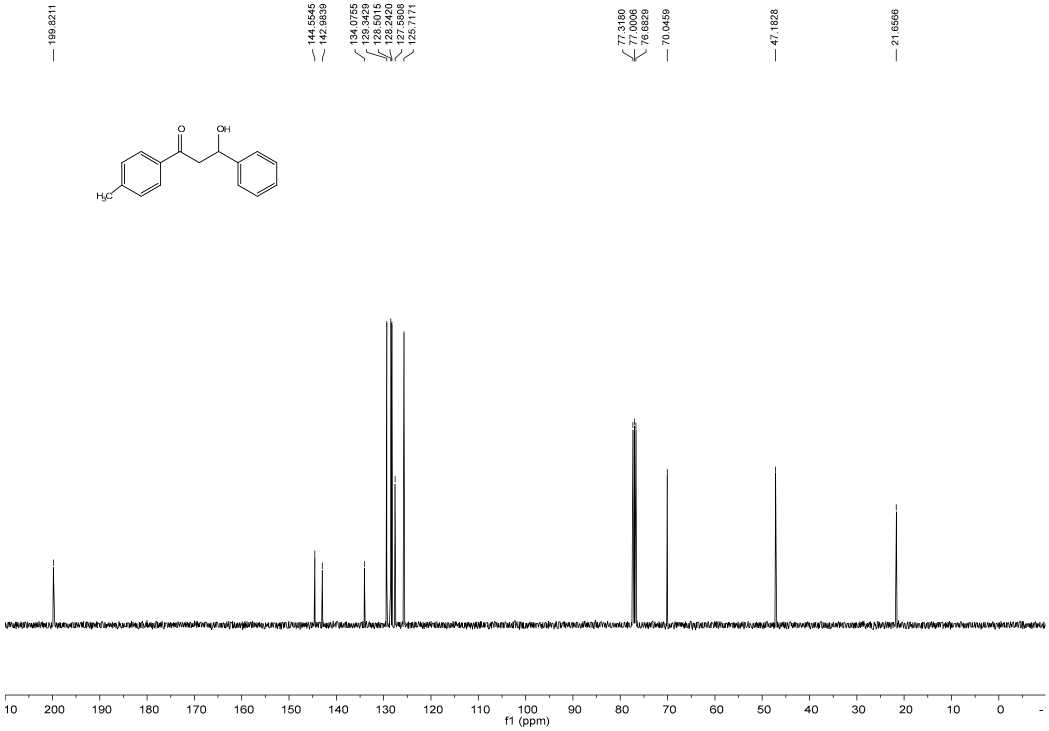
199.8211
144.5545
142.9839
134.0755
129.3429
128.5015
128.2420
127.5808
125.7171
77.3180
77.0006
76.6829
70.0459
47.1828
21.6566
O
OH
H3C
f1 (ppm)

6c

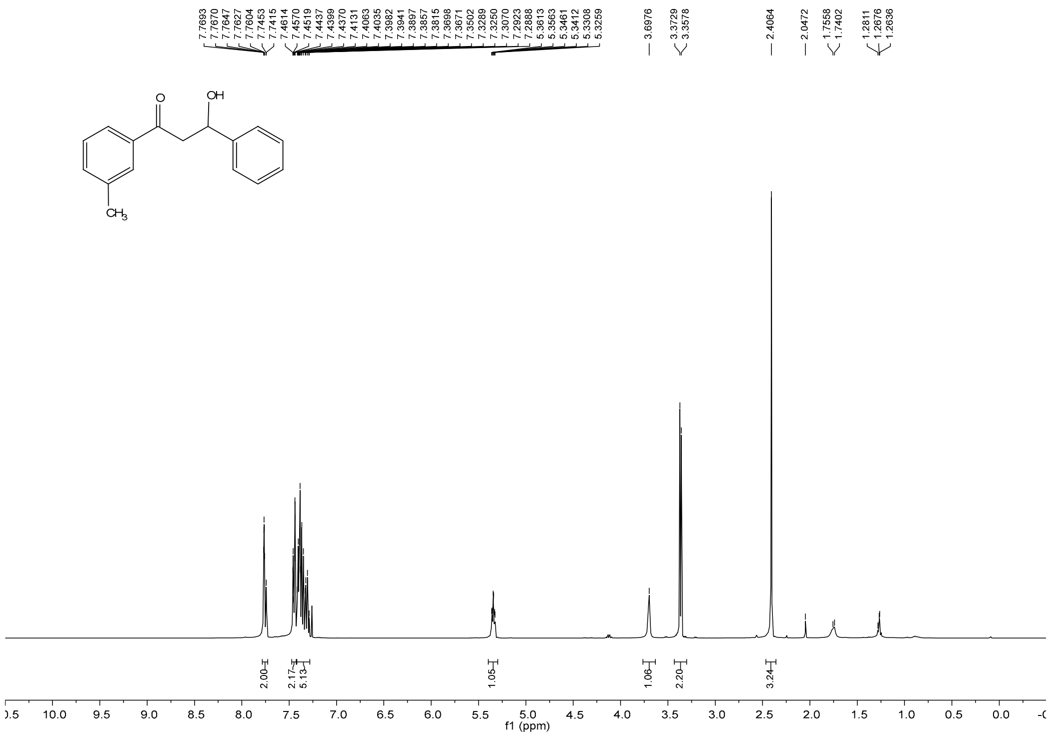
7.7693
7.7670
7.7647
7.7627
7.7604
7.7453
7.7415
7.4614
7.4570
7.4519
7.4437
7.4399
7.4370
7.4131
7.4063
7.4035
7.3982
7.3941
7.3897
7.3857
7.3815
7.3698
7.3671
7.3502
7.3289
7.3250
7.3070
7.2923
7.2888
5.3613
5.3563
5.3461
5.3412
5.3308
5.3259
3.6976
3.3729
3.3578
2.4064
2.0472
1.7558
1.7402
1.2811
1.2676
1.2636
O
OH
CH3
2.00
2.17
5.13
1.05
1.06
2.20
3.24
f1 (ppm)

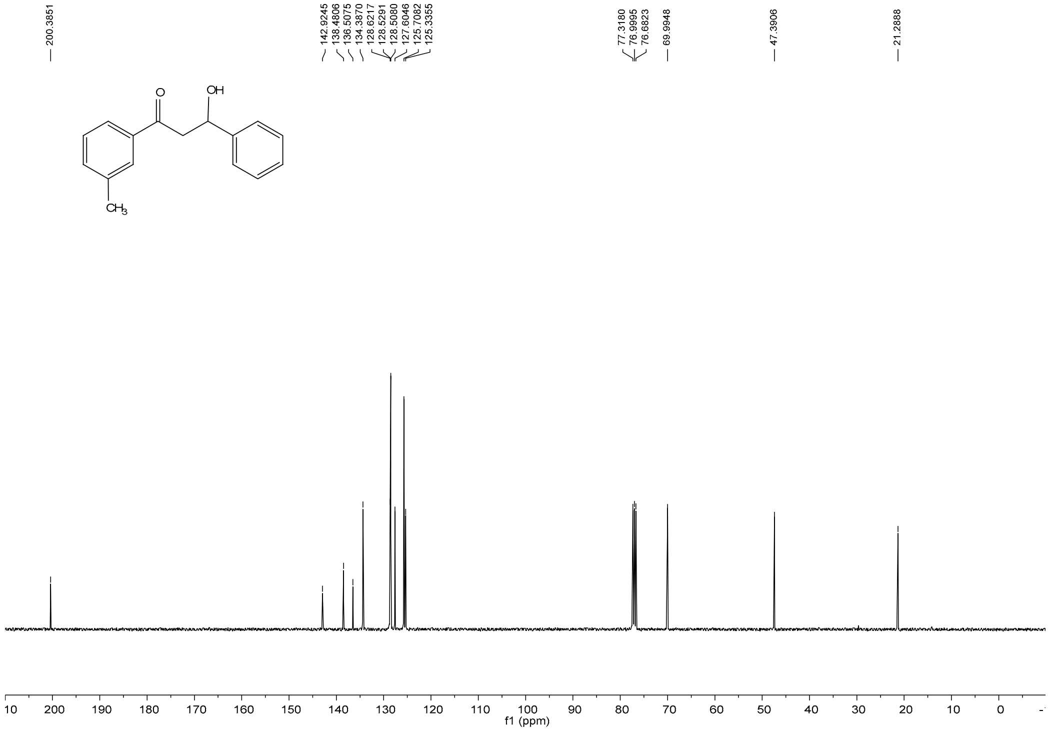
200.3851
142.9245
138.4806
136.5075
134.3870
128.6217
128.5291
128.5080
127.6046
125.7082
125.3355
77.3180
76.9995
76.6823
69.9948
47.3906
21.2888
O
OH
CH3
f1 (ppm)

6d

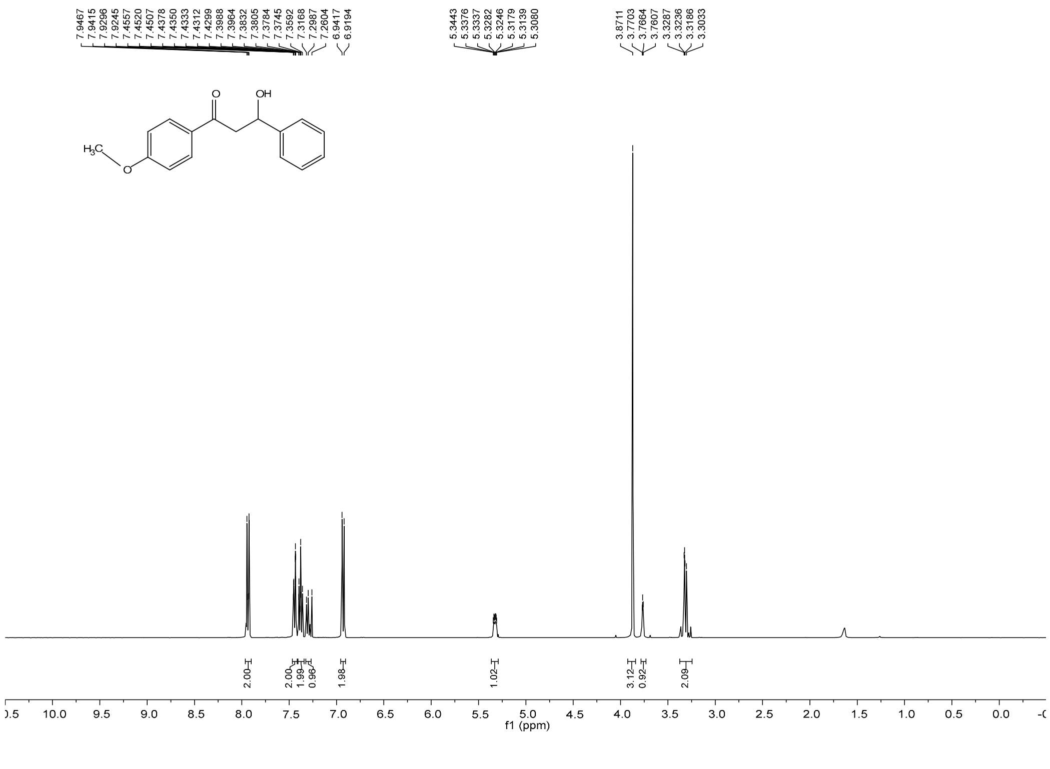
7.9467
7.9415
7.9296
7.9245
7.4557
7.4520
7.4507
7.4378
7.4350
7.4333
7.4312
7.4299
7.3988
7.3964
7.3832
7.3805
7.3784
7.3745
7.3592
7.3168
7.2987
7.2604
6.9417
6.9194
5.3443
5.3376
5.3337
5.3282
5.3246
5.3179
5.3139
5.3080
3.8711
3.7703
3.7664
3.7607
3.3287
3.3236
3.3186
3.3033
O
OH
H3C
O
2.00
2.00
1.99
0.96
1.98
1.02
3.12
0.92
2.09
f1 (ppm)

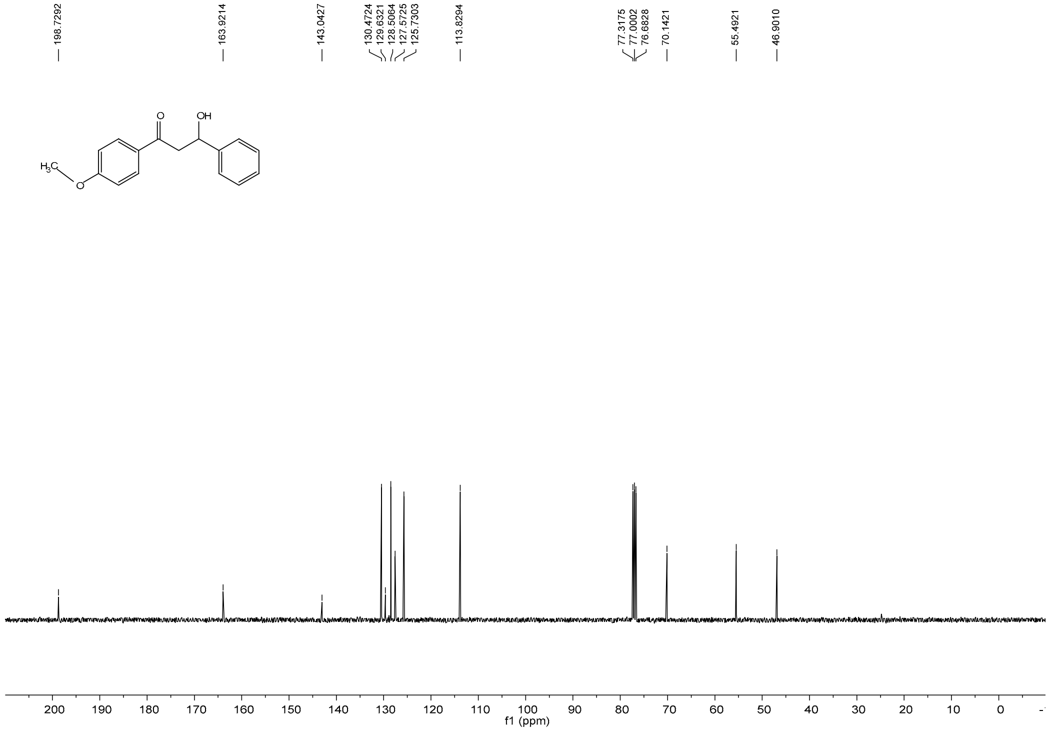
198.7292
163.9214
143.0427
130.4724
129.6321
128.5064
127.5725
125.7303
113.8294
77.3175
77.0002
76.6828
70.1421
55.4921
46.9010
OH
O
f1 (ppm)

6e.

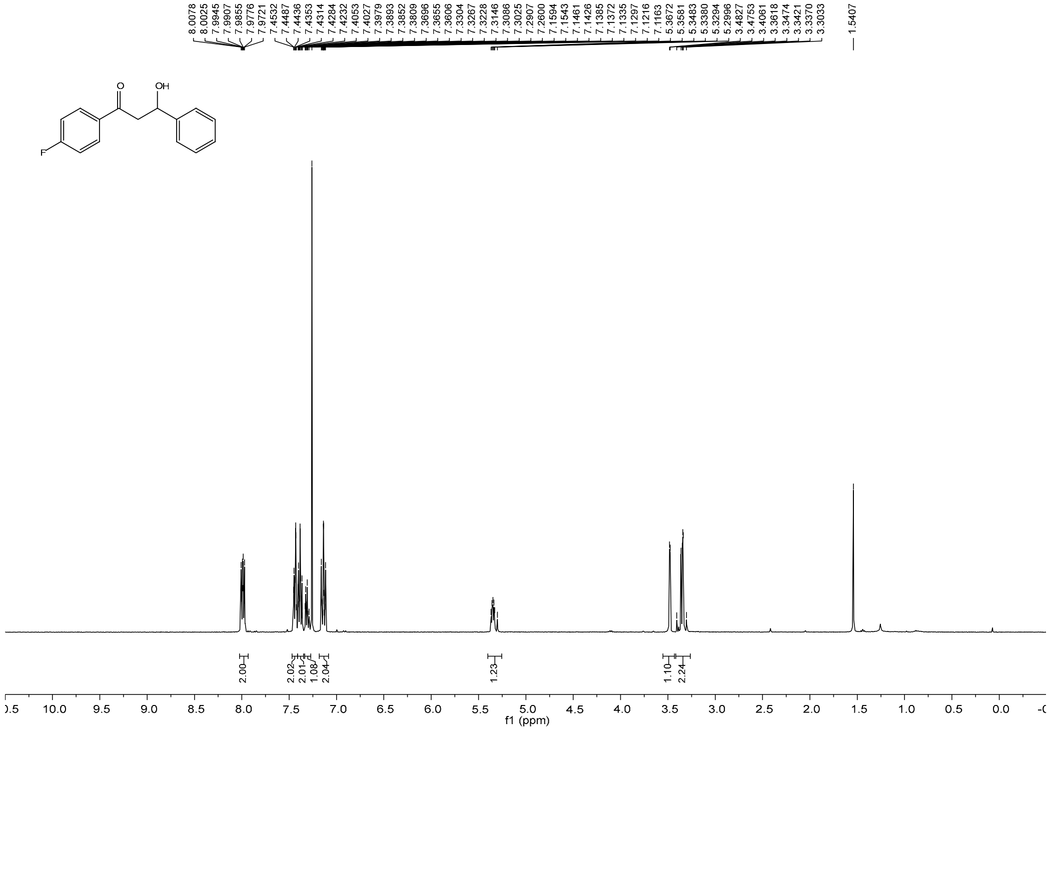
8.0078
8.0025
7.9945
7.9907
7.9855
7.9776
7.9721
7.4532
7.4487
7.4436
7.4353
7.4314
7.4284
7.4232
7.4053
7.4027
7.3979
7.3893
7.3852
7.3809
7.3696
7.3655
7.3606
7.3304
7.3267
7.3228
7.3146
7.3086
7.3025
7.2907
7.2600
7.1594
7.1543
7.1461
7.1426
7.1385
7.1372
7.1335
7.1297
7.1216
7.1163
5.3672
5.3581
5.3483
5.3380
5.3294
5.2996
3.4827
3.4753
3.4061
3.3618
3.3474
3.3421
3.3370
3.3033
1.5407
O
OH
F
2.00
2.02
2.01
1.08
2.04
1.23
1.10
2.24
f1 (ppm)

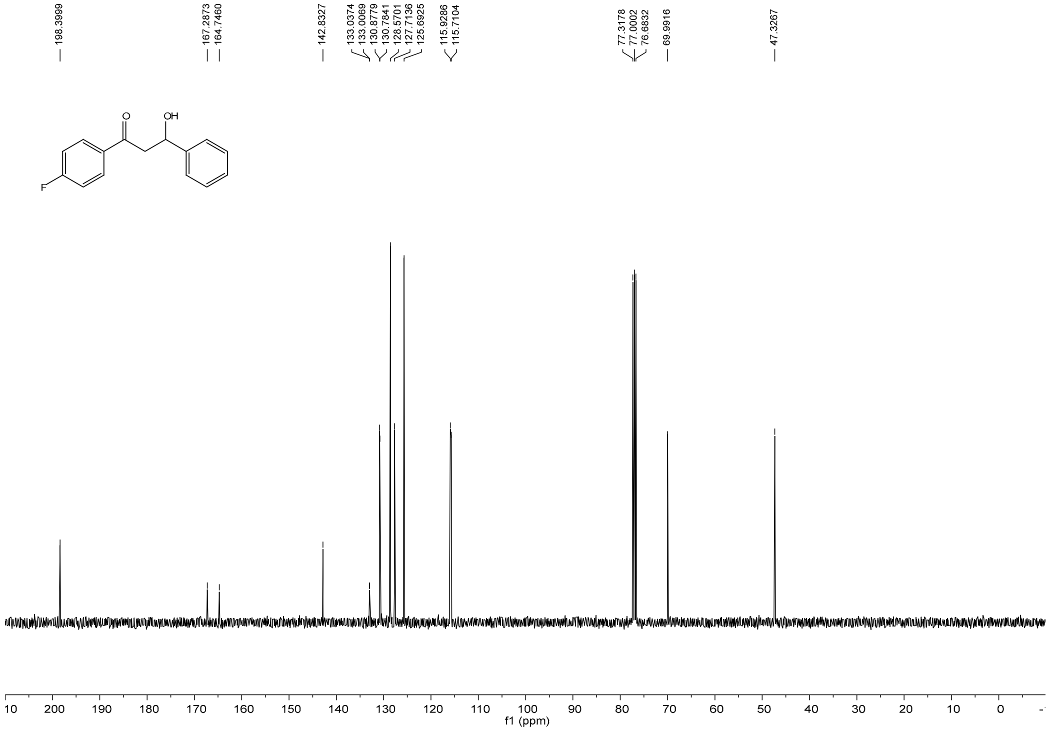
198.3999
167.2873
164.7460
142.8327
133.0374
133.0069
130.8779
130.7841
128.5701
127.7136
125.6925
115.9286
115.7104
77.3178
77.0002
76.6832
69.9916
47.3267
O
OH
F
f1 (ppm)

6f

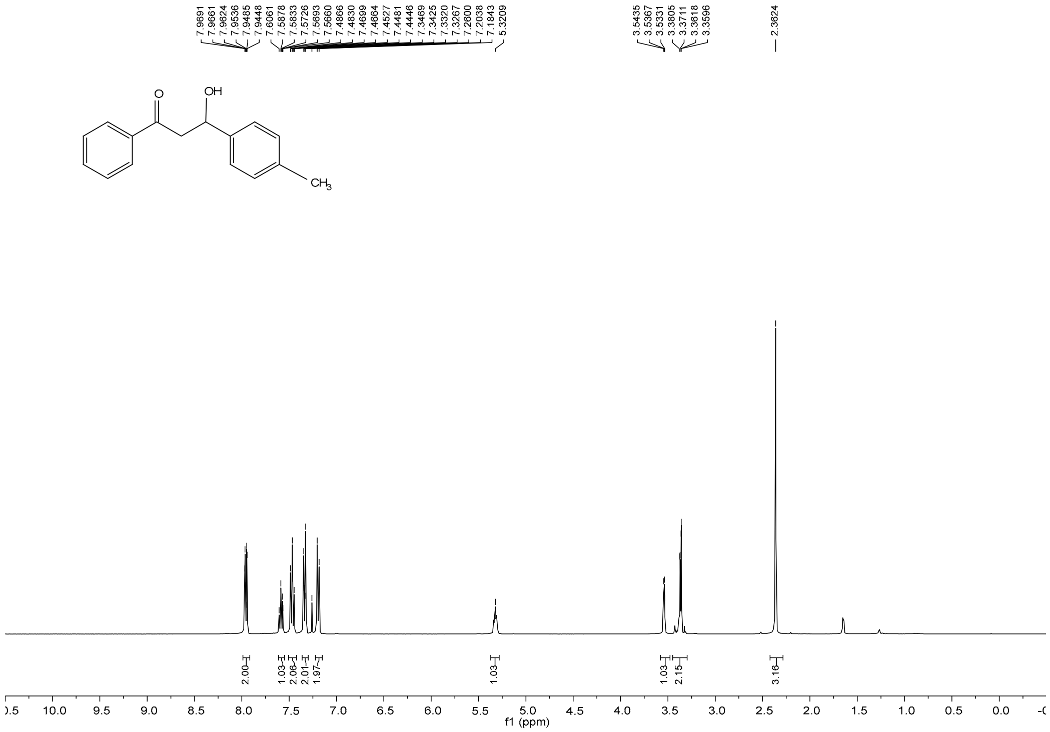
7.9691
7.9661
7.9624
7.9536
7.9485
7.9448
7.6061
7.5878
7.5833
7.5726
7.5693
7.5660
7.4866
7.4830
7.4699
7.4664
7.4527
7.4481
7.4446
7.3469
7.3425
7.3320
7.3267
7.2600
7.2038
7.1843
5.3209
3.5435
3.5387
3.5331
3.3805
3.3711
3.3618
3.3596
2.3624
O
OH
CH3
2.00
1.03
2.06
2.01
1.97
1.03
1.03
2.15
3.16
f1 (ppm)

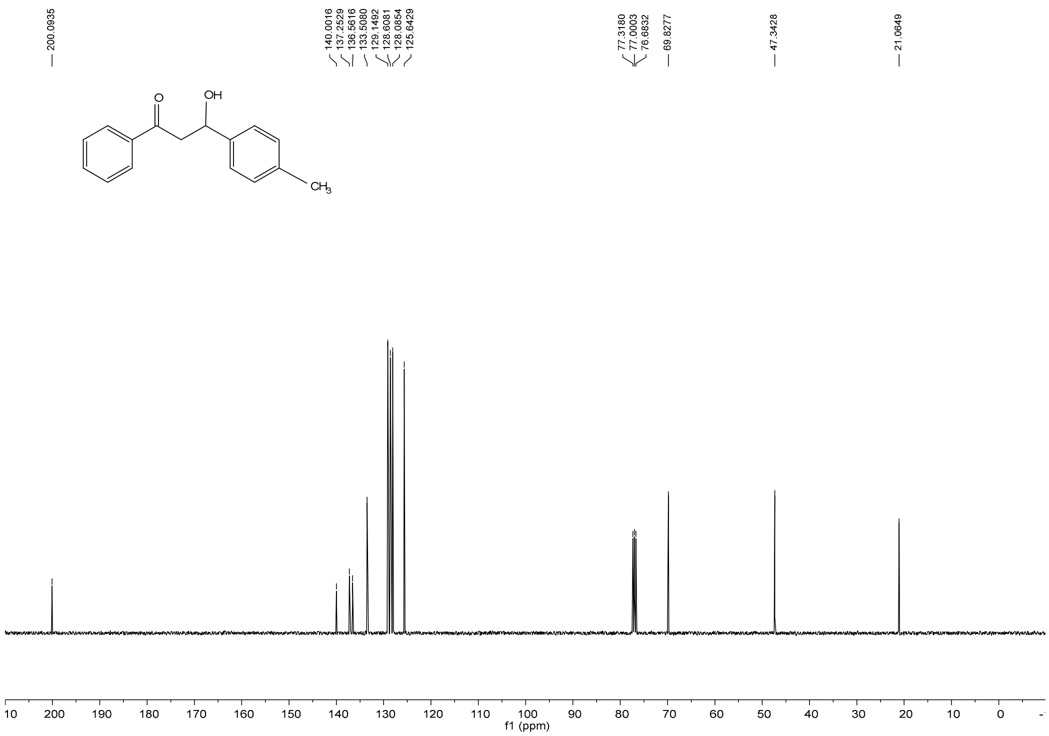
200.0935
140.0016
137.2529
136.5616
133.5080
129.1492
128.6081
128.0854
125.6429
77.3180
77.0003
76.6832
69.8277
47.3428
21.0649
OH
O
CH3
f1 (ppm)

6g

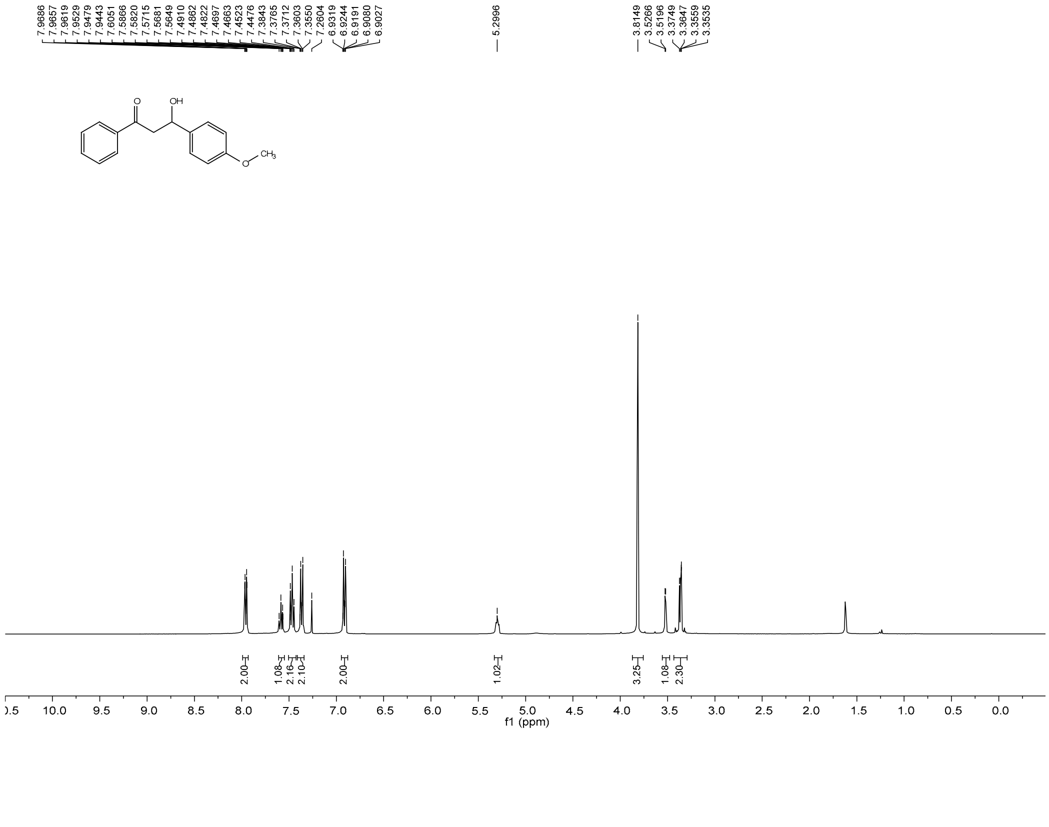
7.9686
7.9657
7.9619
7.9629
7.9479
7.9443
7.6051
7.5866
7.5820
7.5715
7.5681
7.5649
7.4910
7.4862
7.4822
7.4697
7.4663
7.4523
7.4476
7.3843
7.3765
7.3712
7.3603
7.3550
7.2604
6.9319
6.9244
6.9191
6.9080
6.9027
5.2996
3.8149
3.5266
3.5196
3.3749
3.3647
3.3559
3.3535
O
OH
CH3
2.00
1.08
2.16
2.10
2.00
1.02
3.25
1.08
2.30
f1 (ppm)

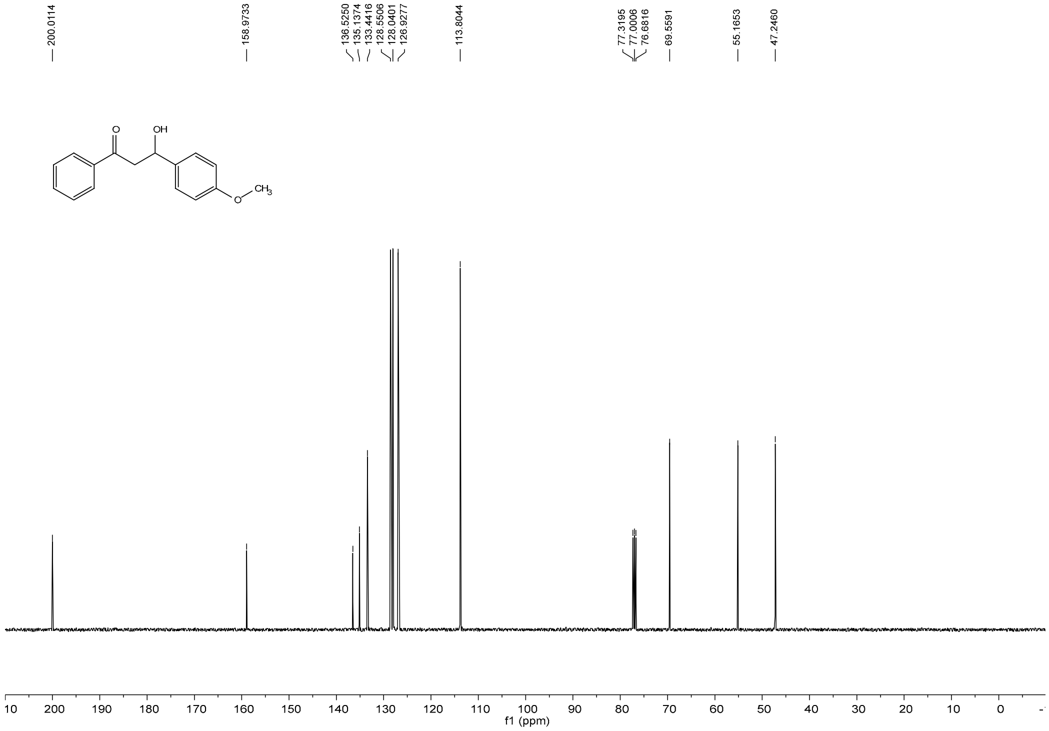
200.0114
158.9733
136.5250
135.1374
133.4416
128.5506
128.0401
126.9277
113.8044
77.3195
77.0006
76.6816
69.5591
55.1653
47.2460
O
OH
O
CH3
f1 (ppm)

6h

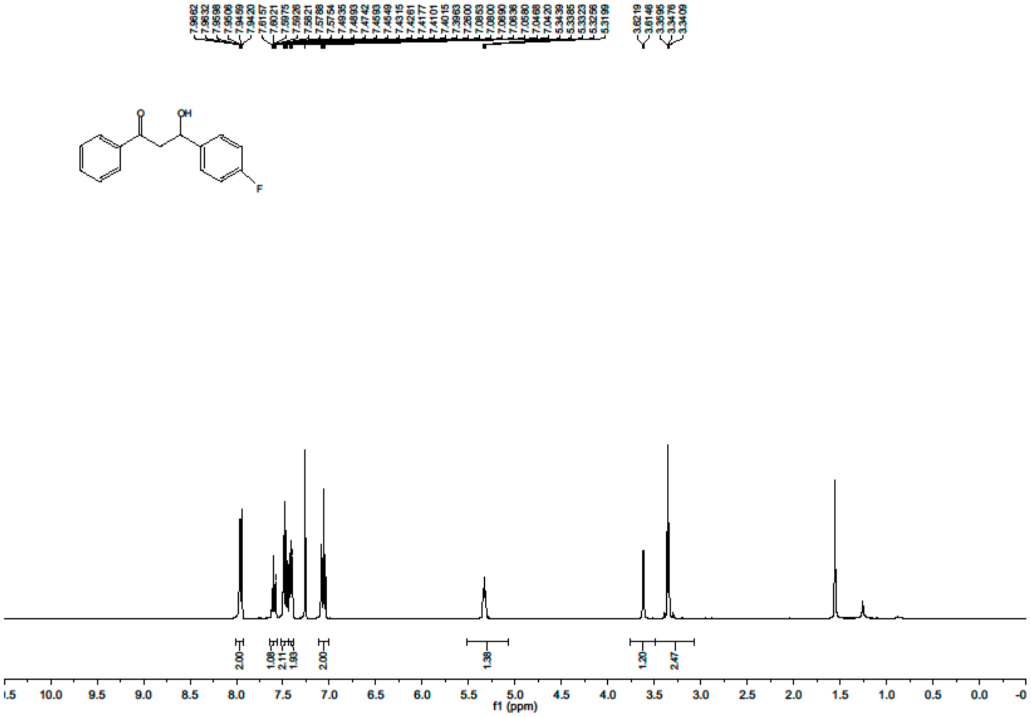
O
OH
F
2.00
1.08
2.11
1.93
2.00
1.38
1.20
2.47
f1 (ppm)

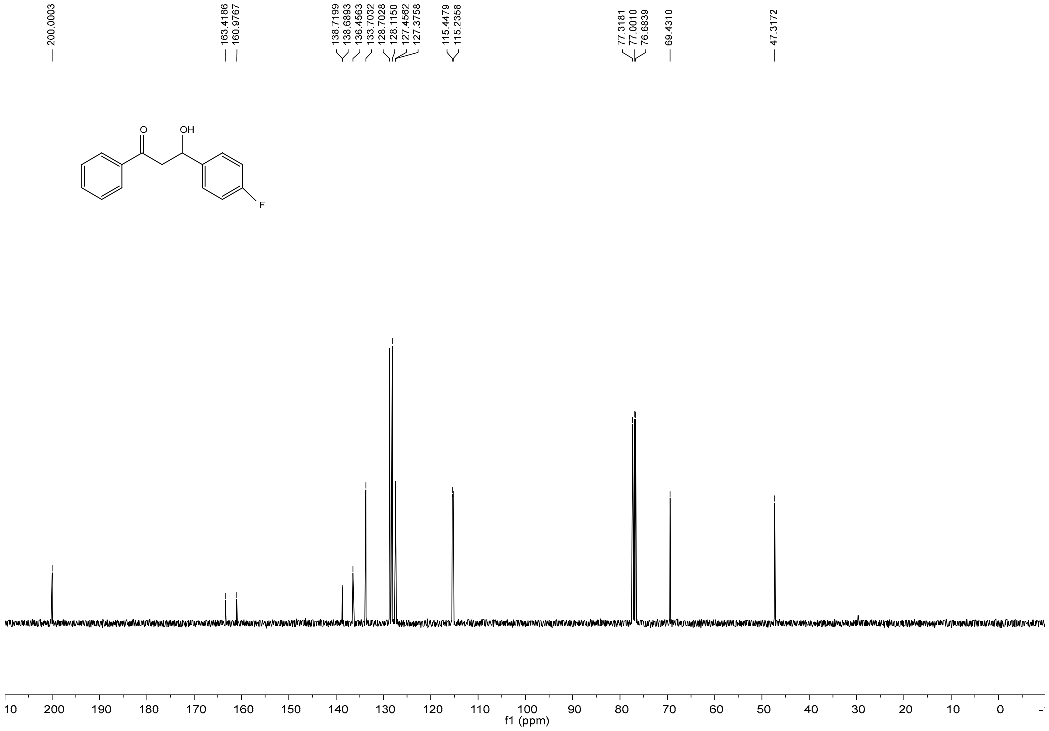

6i

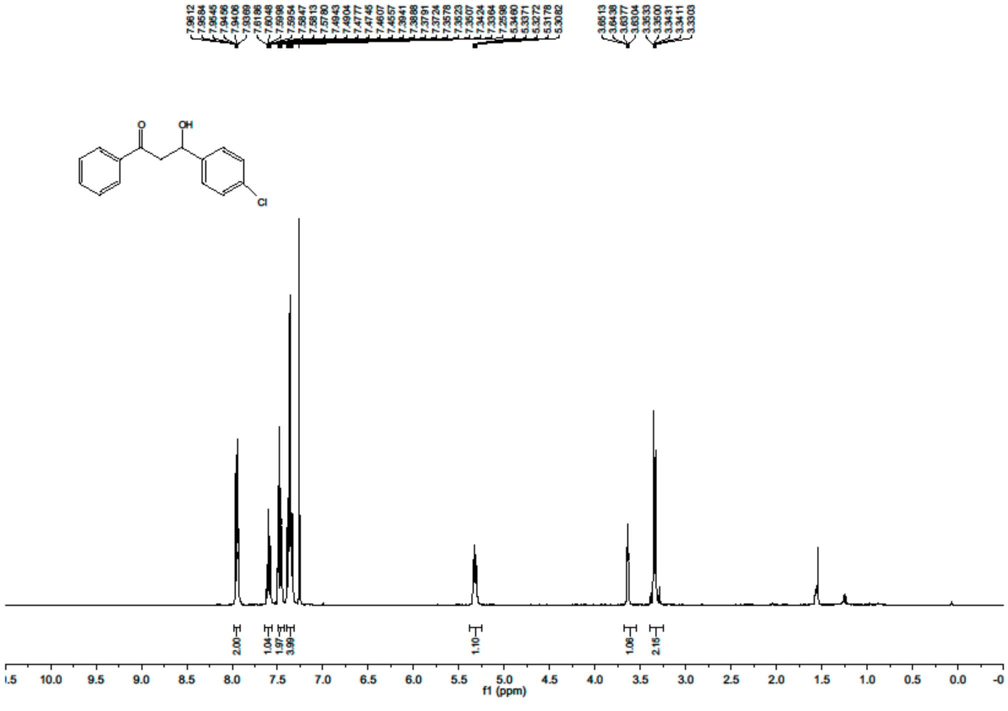

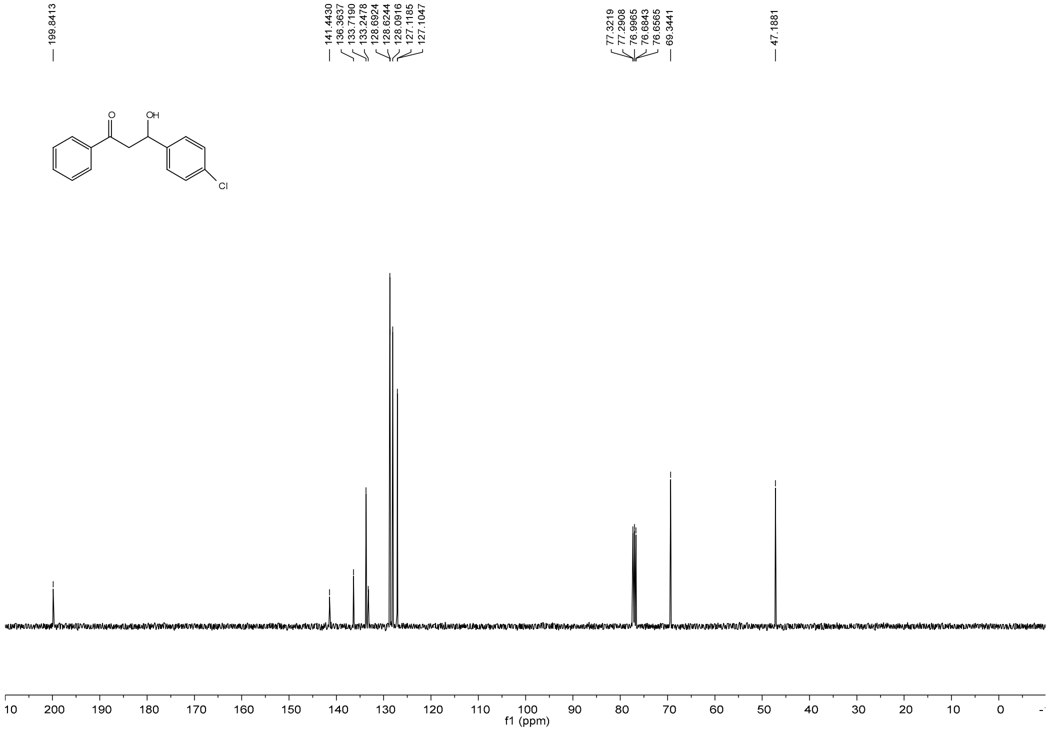
199.8413
141.4430
136.3637
133.7190
133.2478
128.6924
128.6244
128.0916
127.1185
127.1047
77.3219
77.2908
76.9965
76.6843
76.6565
69.3441
47.1881
O
OH
Cl
f1 (ppm)

6j

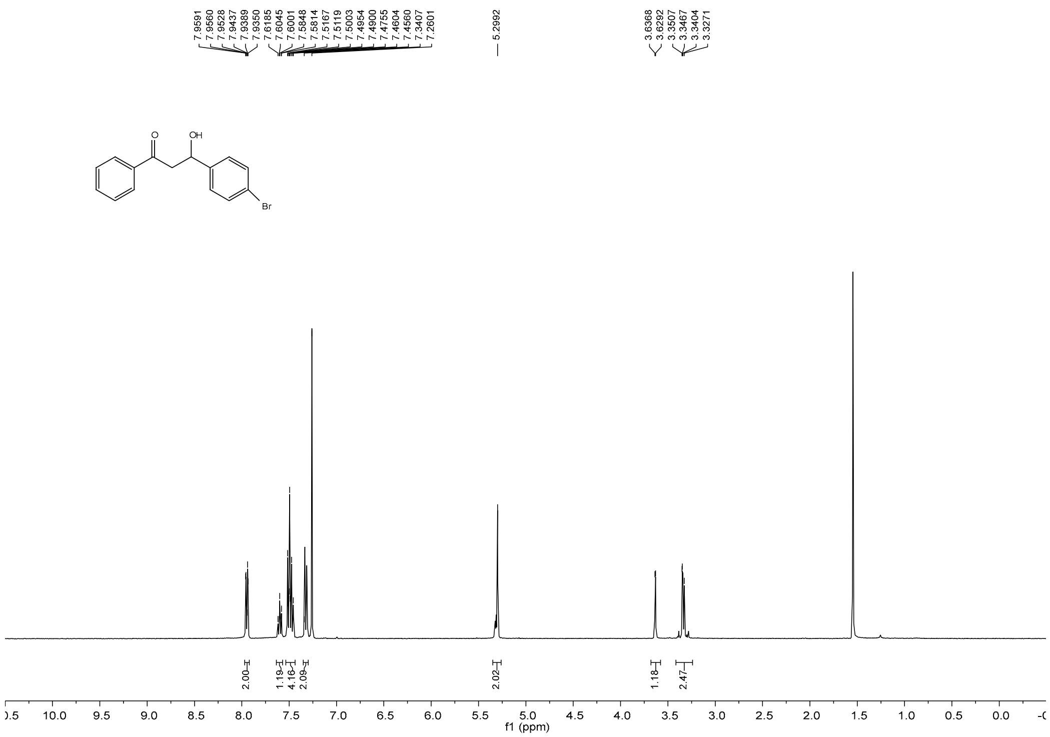
7.9591
7.9560
7.9528
7.9437
7.9389
7.9350
7.6185
7.6045
7.6001
7.5848
7.5814
7.5167
7.5119
7.5003
7.4954
7.4900
7.4755
7.4604
7.4560
7.3407
7.2601
5.2992
3.6368
3.6292
3.3507
3.3467
3.3404
3.3271
O
OH
Br
2.00
1.19
4.16
2.09
2.02
1.18
2.47
f1 (ppm)

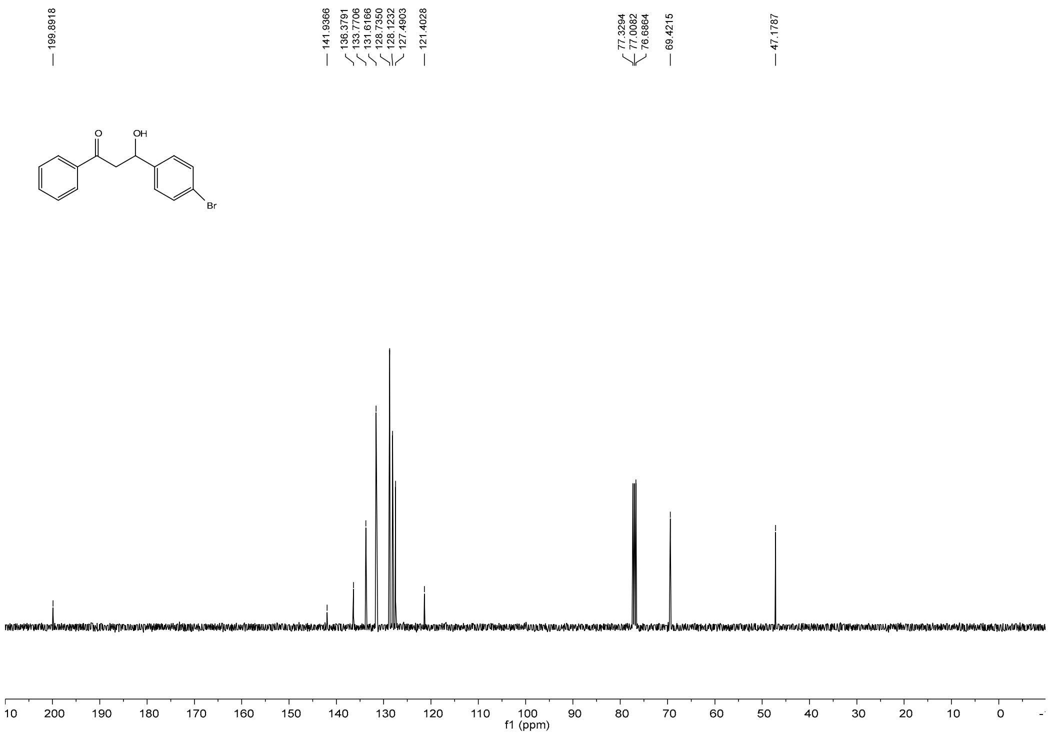
199.8918
141.9366
136.3791
133.7706
131.6166
128.7350
128.1232
127.4903
121.4028
77.3294
77.0082
76.6884
69.4215
47.1787
OH
O
Br
f1 (ppm)

6k

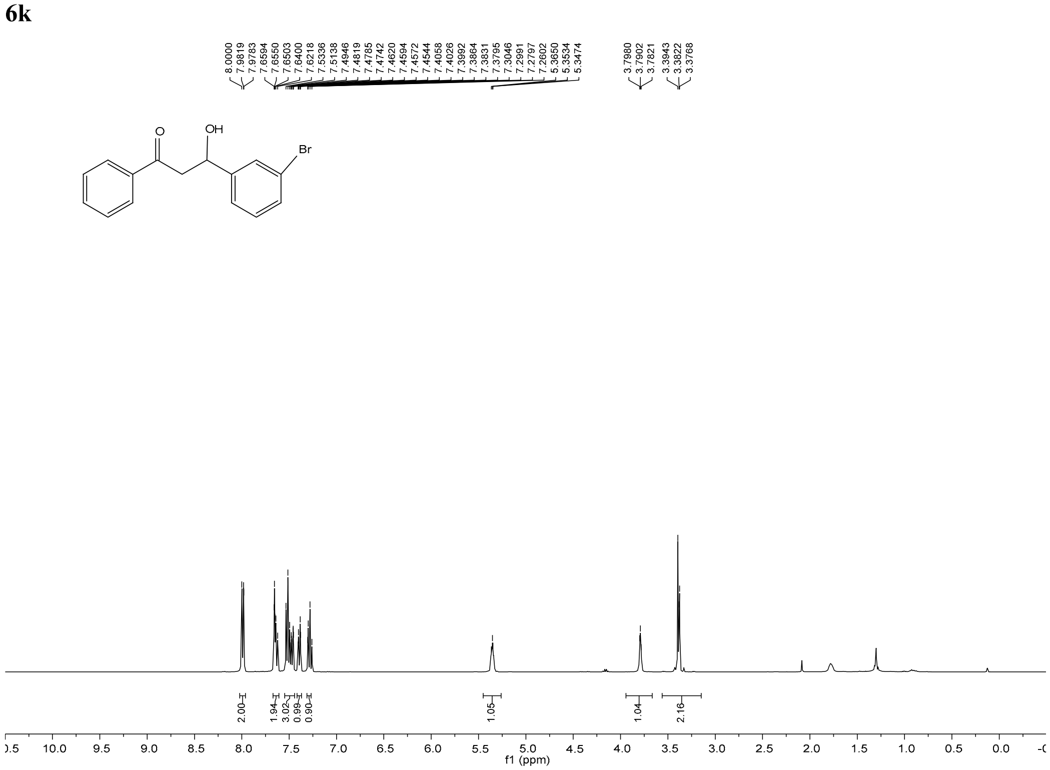
8.0000
7.9819
7.9783
7.6594
7.6550
7.6503
7.6400
7.6218
7.5336
7.5138
7.4946
7.4819
7.4785
7.4742
7.4620
7.4594
7.4572
7.4544
7.4058
7.4026
7.3992
7.3864
7.3831
7.3795
7.3046
7.2991
7.2797
7.2602
5.3650
5.3534
5.3474
3.7980
3.7902
3.7821
3.3943
3.3822
3.3768
O
OH
Br
2.00
1.94
3.02
0.99
0.90
1.05
1.04
2.16
f1 (ppm)

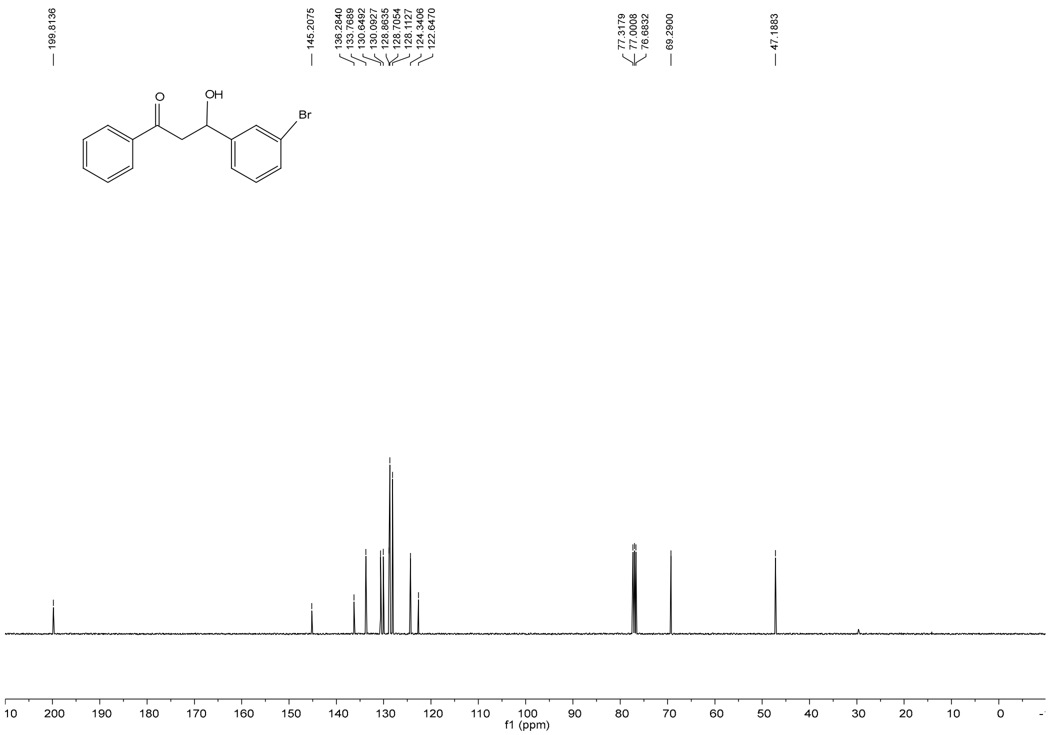
199.8136
145.2075
136.2840
133.7689
130.6492
130.0927
128.8635
128.7054
128.1127
124.3406
122.6470
77.3179
77.0008
76.6832
69.2900
47.1883
OH
O
Br
f1 (ppm)

6l

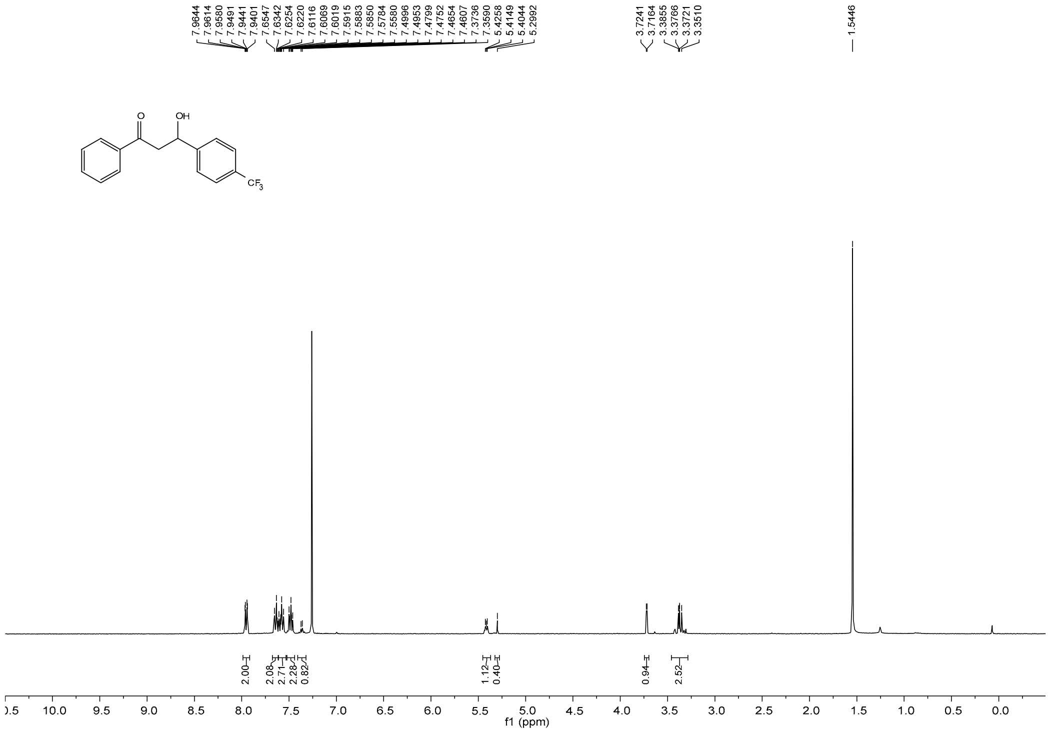
7.9644
7.9614
7.9580
7.9491
7.9441
7.9401
7.6547
7.6342
7.6254
7.6220
7.6116
7.6069
7.6019
7.5915
7.5883
7.5850
7.5784
7.5580
7.4996
7.4953
7.4799
7.4752
7.4654
7.4607
7.3736
7.3590
5.4258
5.4149
5.4044
5.2992
3.7241
3.7164
3.3855
3.3766
3.3721
3.3510
1.5446
O
OH
CF_3
2.00
2.08
2.71
2.28
0.82
1.12
0.40
0.94
2.52
f1 (ppm)

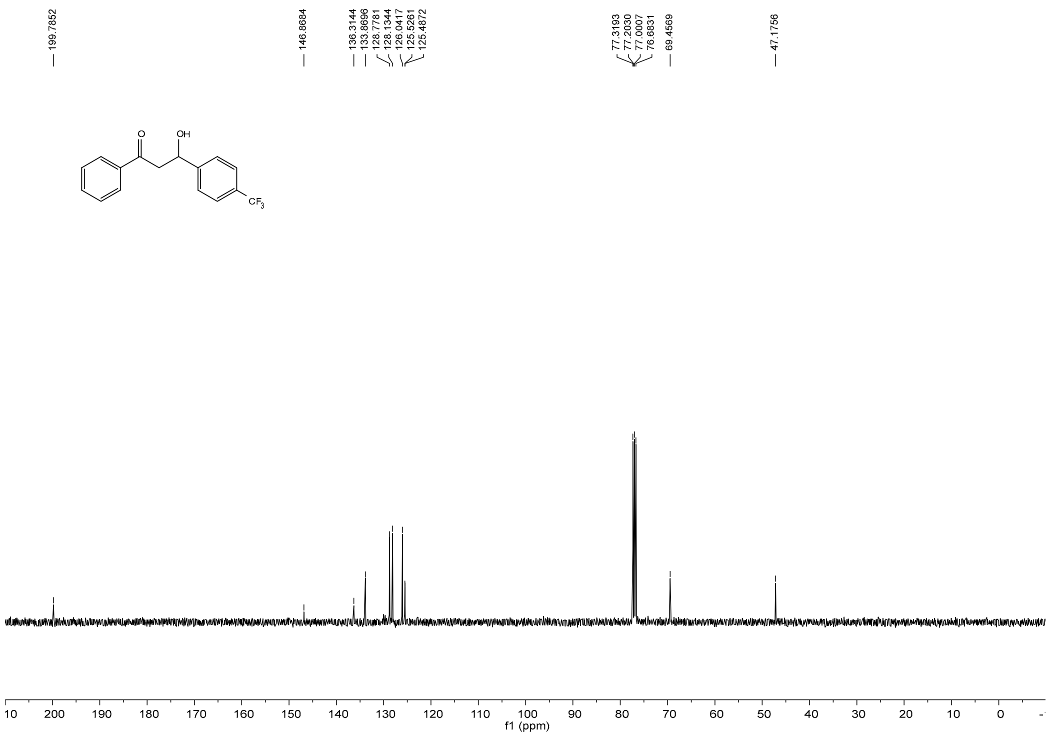

6m

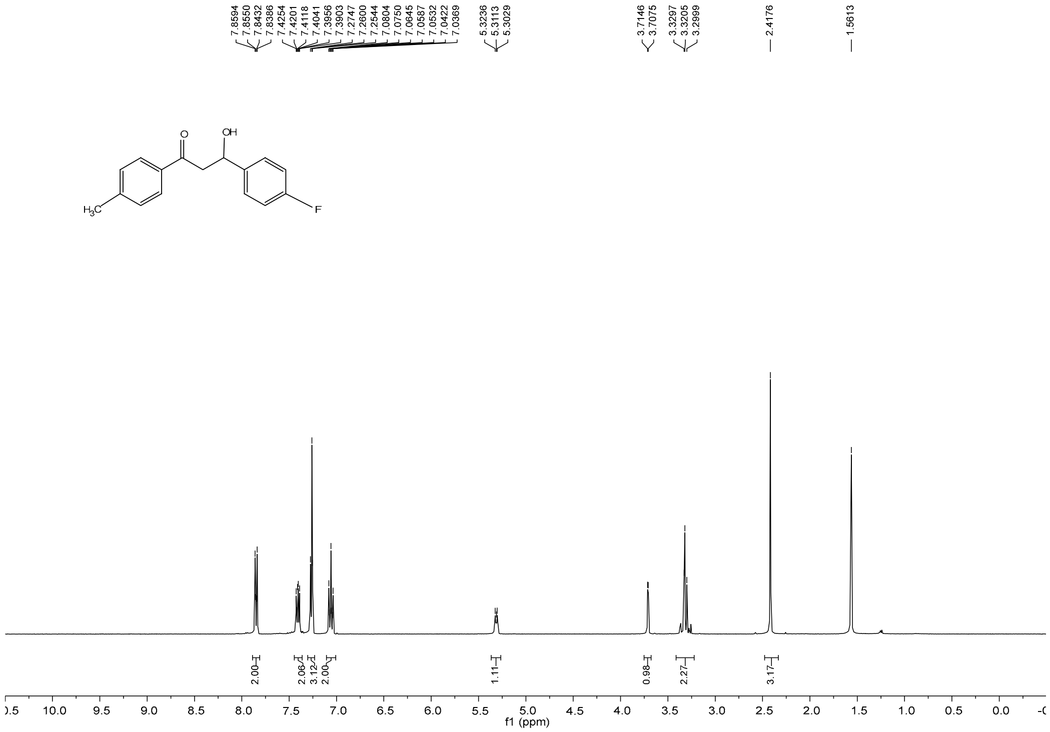

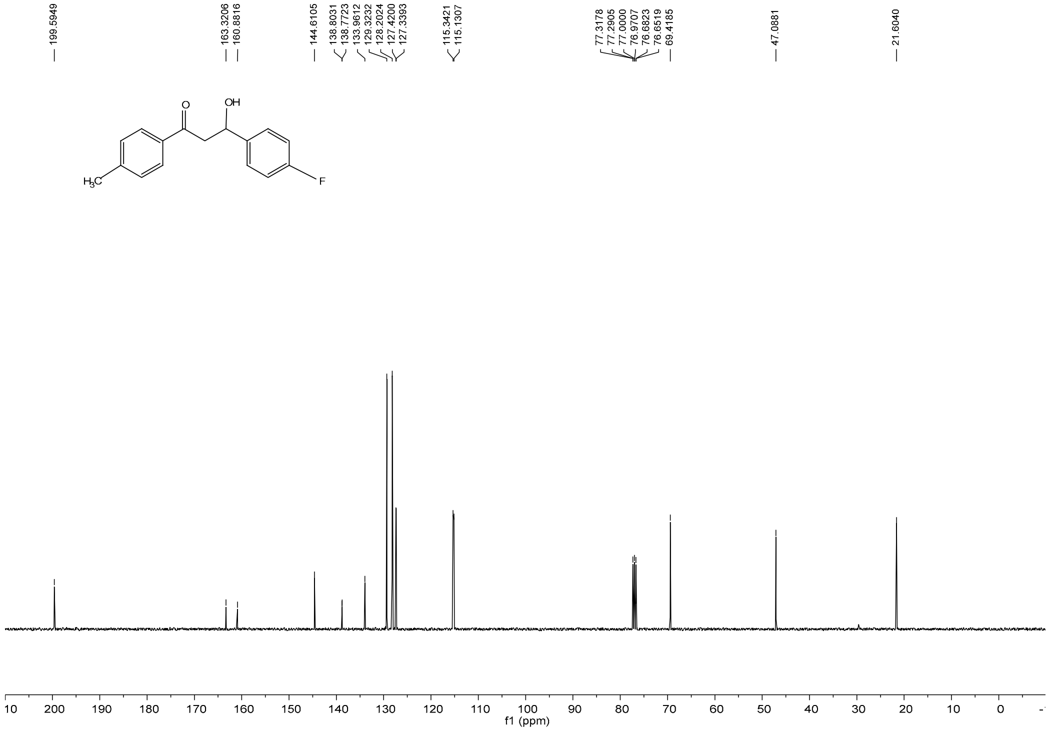
199.5949
163.3206
160.8816
144.6105
138.8031
138.7723
133.9612
129.3232
128.2024
127.4200
127.3393
115.3421
115.1307
77.3178
77.2905
77.0000
76.9707
76.6823
76.6519
69.4185
47.0881
21.6040
O
OH
H3C
F
f1 (ppm)

6n

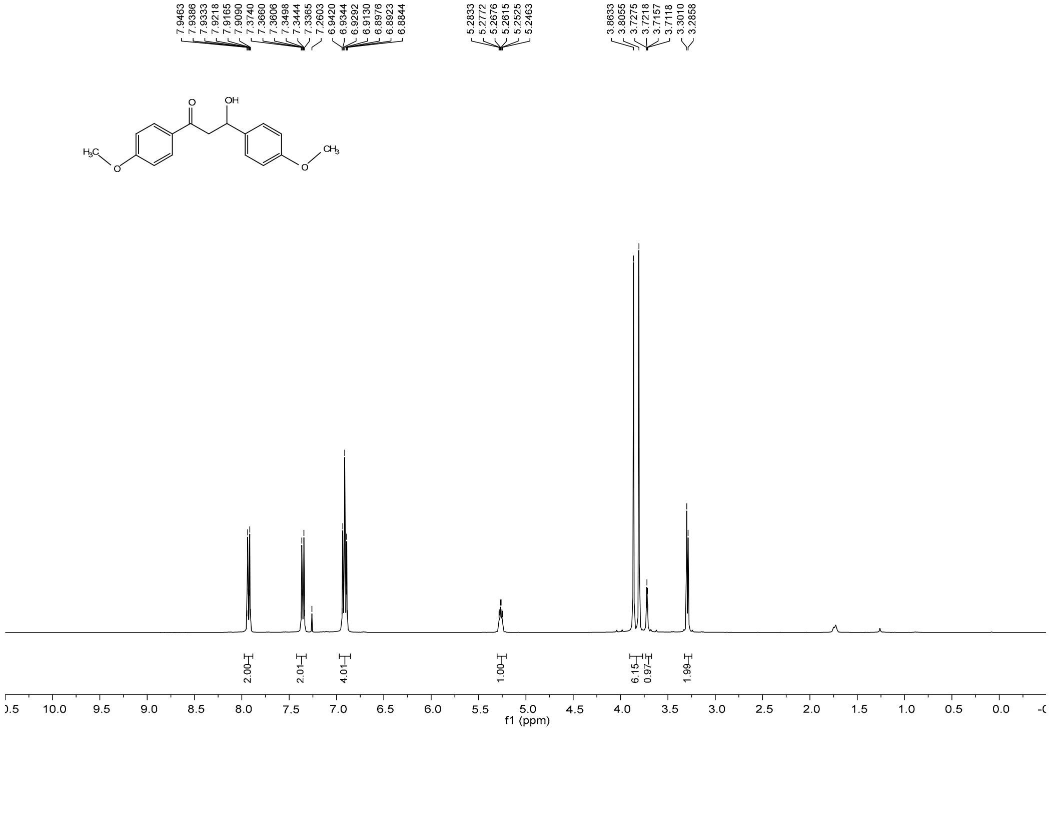
7.9463
7.9386
7.9333
7.9218
7.9165
7.9090
7.3740
7.3660
7.3606
7.3498
7.3444
7.3365
7.2603
6.9420
6.9344
6.9292
6.9130
6.8976
6.8923
6.8844
5.2833
5.2772
5.2676
5.2615
5.2525
5.2463
3.8633
3.8055
3.7275
3.7218
3.7157
3.7118
3.3010
3.2858
O
OH
H3C
CH3
O
O
2.00
2.01
4.01
1.00
6.15
0.97
1.99
f1 (ppm)

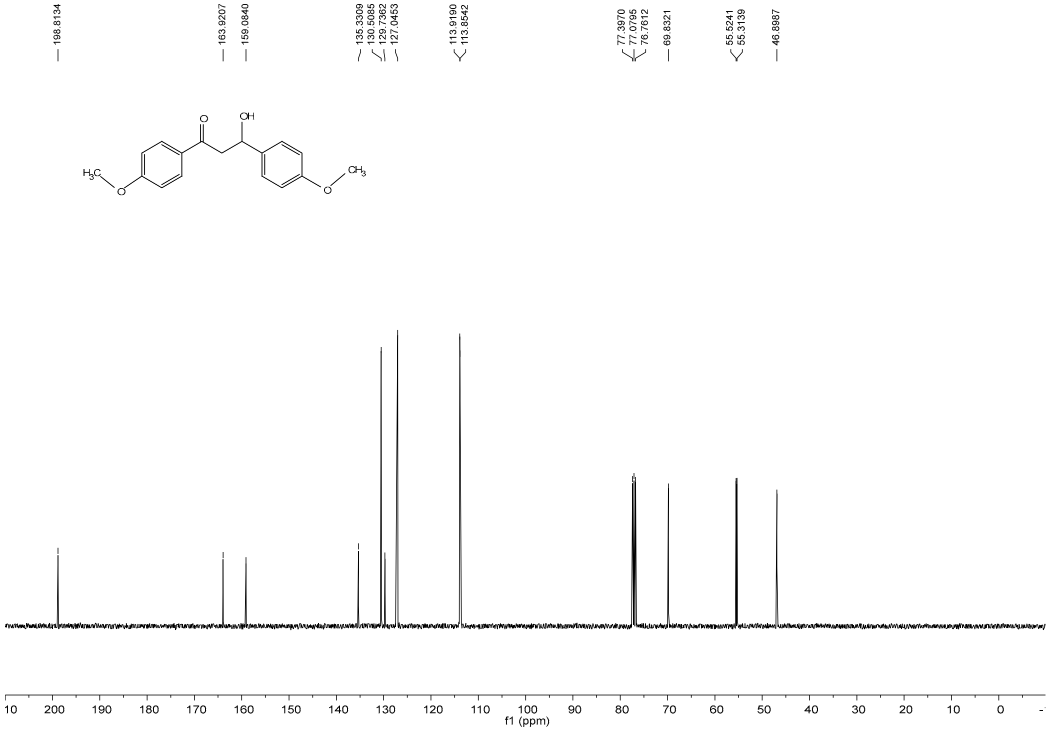
198.8134
163.9207
159.0840
135.3309
130.5085
129.7362
127.0453
113.9190
113.8542
77.3970
77.0795
76.7612
69.8321
55.5241
55.3139
46.8987
OH
O
H3C
O
O
CH3
f1 (ppm)

6o

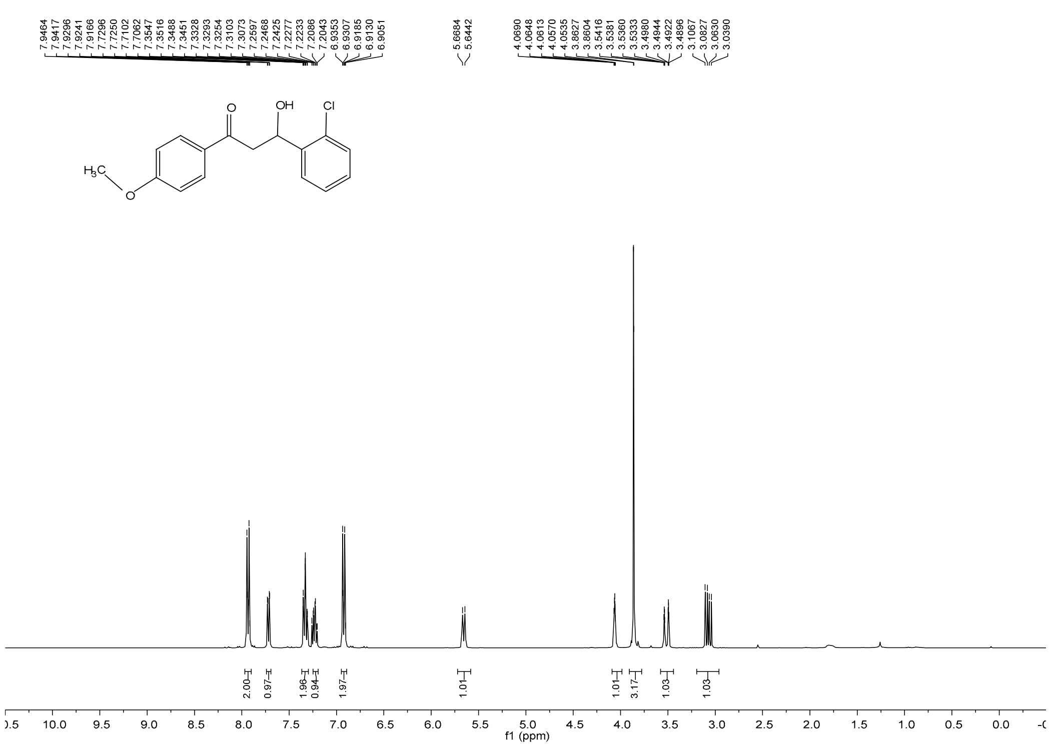
7.9464
7.9417
7.9296
7.9241
7.9166
7.7296
7.7250
7.7102
7.7062
7.3547
7.3516
7.3488
7.3451
7.3328
7.3293
7.3254
7.3103
7.3073
7.2597
7.2468
7.2425
7.2277
7.2233
7.2086
7.2043
6.9353
6.9307
6.9185
6.9130
6.9051
5.6684
5.6442
4.0690
4.0648
4.0613
4.0570
4.0535
3.8627
3.8604
3.5416
3.5381
3.5360
3.5333
3.4980
3.4944
3.4922
3.4896
3.1067
3.0827
3.0630
3.0390
O
OH
Cl
H3C
O
2.00
0.97
1.96
0.94
1.97
1.01
1.01
3.17
1.03
1.03
f1 (ppm)

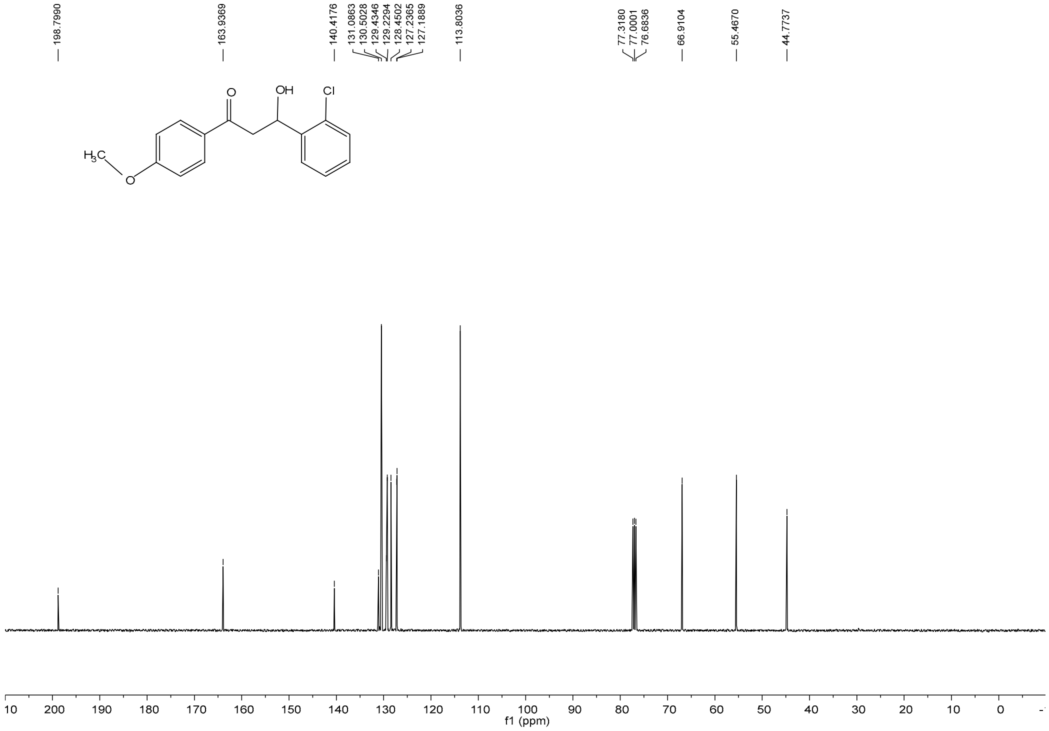
198.7990
163.9369
140.4176
131.0863
130.5028
129.4346
129.2294
128.4502
127.2365
127.1889
113.8036
77.3180
77.0001
76.6836
66.9104
55.4670
44.7737
O
OH
Cl
H3C
O
f1 (ppm)

6p

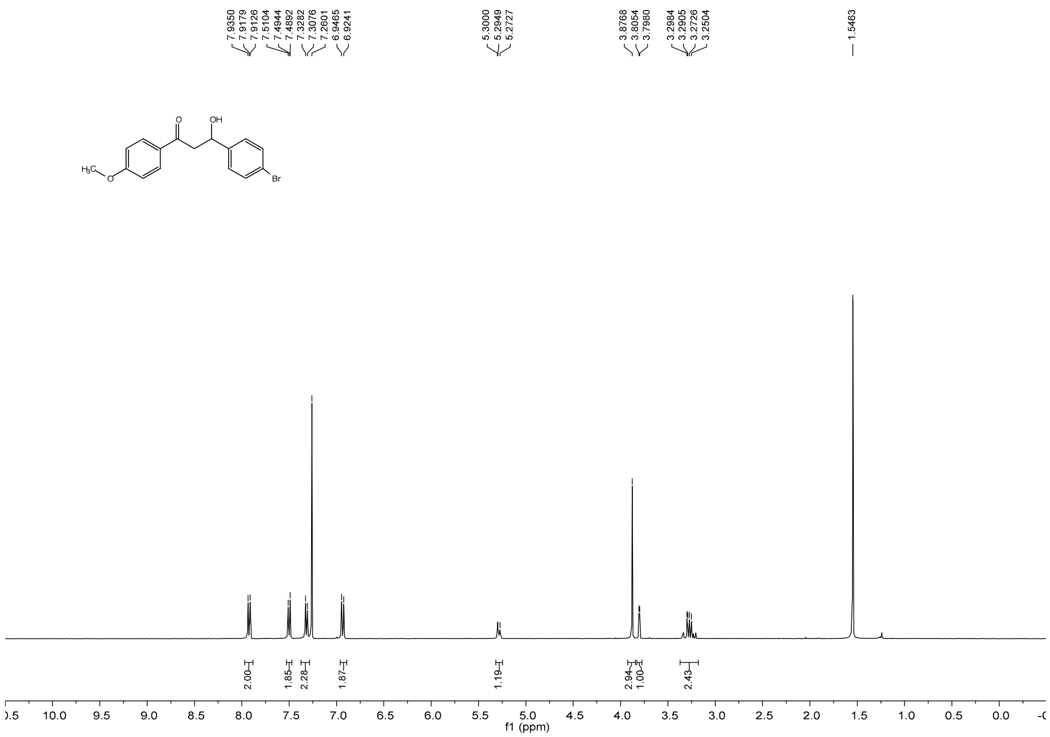
7.9350
7.9179
7.9126
7.5104
7.4944
7.4892
7.3282
7.3076
7.2601
6.9465
6.9241
5.3000
5.2949
5.2727
3.8768
3.8054
3.7980
3.2984
3.2905
3.2726
3.2504
1.5463
O
OH
H3C
O
Br
2.00
1.85
2.28
1.87
1.19
2.94
1.00
2.43
f1 (ppm)

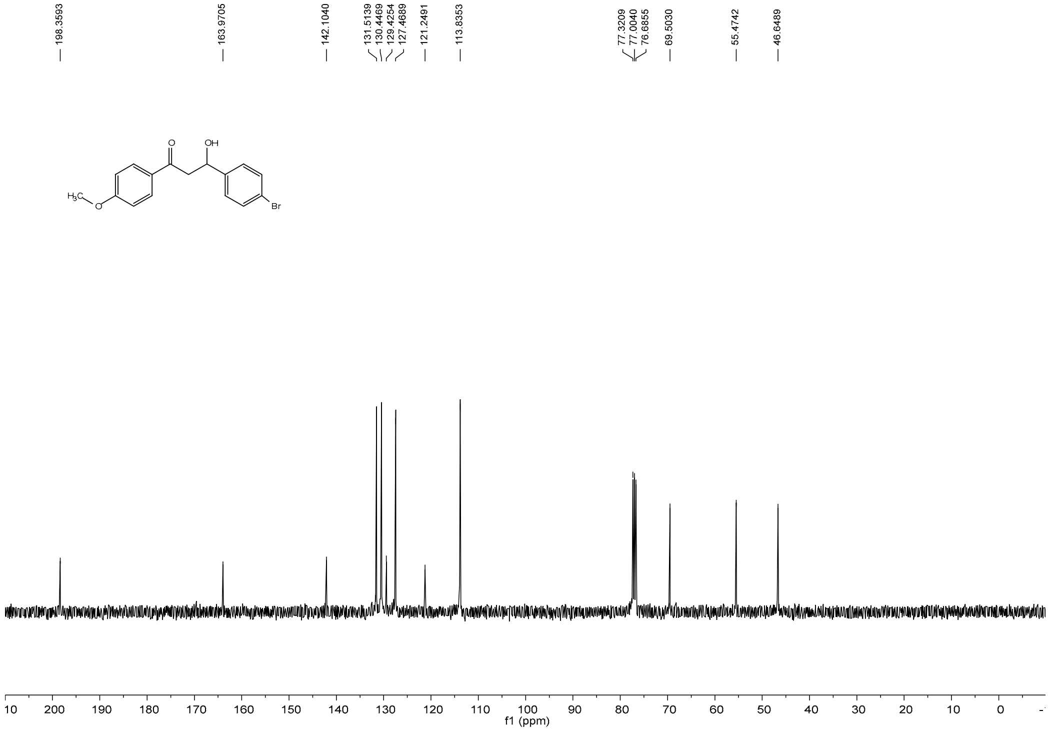
198.3593
163.9705
142.1040
131.5139
130.4469
129.4254
127.4689
121.2491
113.8353
77.3209
77.0040
76.6855
69.5030
55.4742
46.6489
O
OH
H_3C
O
Br
f1 (ppm)

6q

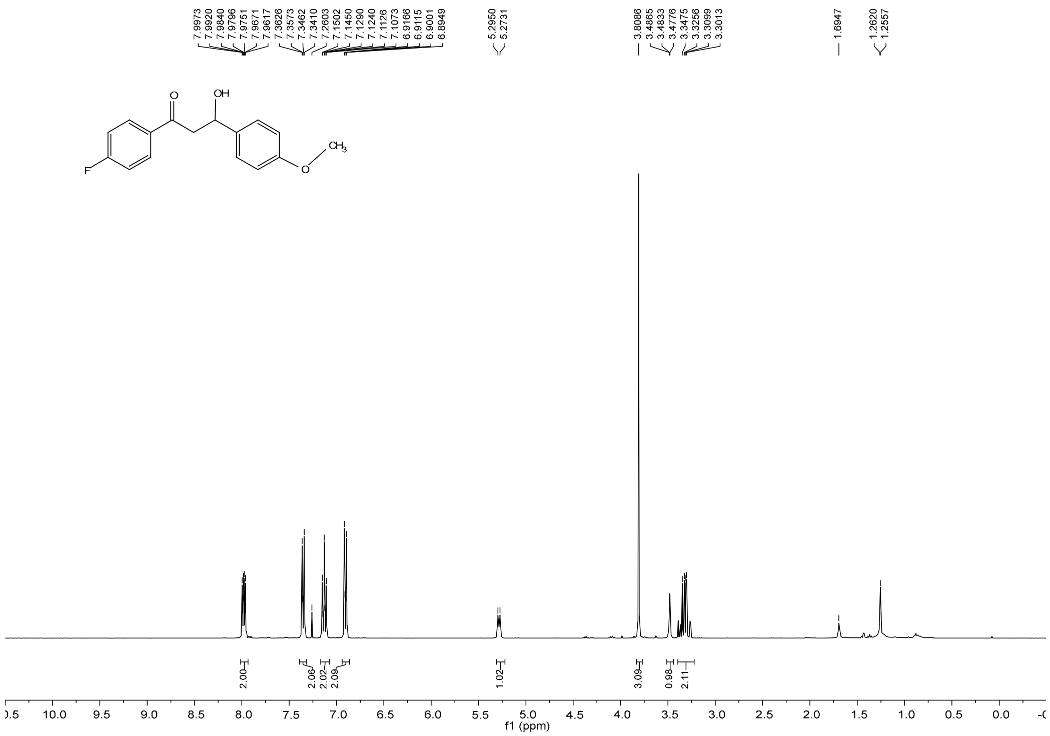
7.9973
7.9920
7.9840
7.9796
7.9751
7.9671
7.9617
7.3626
7.3573
7.3462
7.3410
7.2603
7.1502
7.1450
7.1290
7.1240
7.1126
7.1073
6.9166
6.9115
6.9001
6.8949
5.2950
5.2731
3.8086
3.4865
3.4833
3.4776
3.3475
3.3256
3.3099
3.3013
1.6947
1.2620
1.2557
O
OH
F
O
CH_3
2.00
2.06
2.02
2.09
1.02
3.09
0.98
2.11
f1 (ppm)

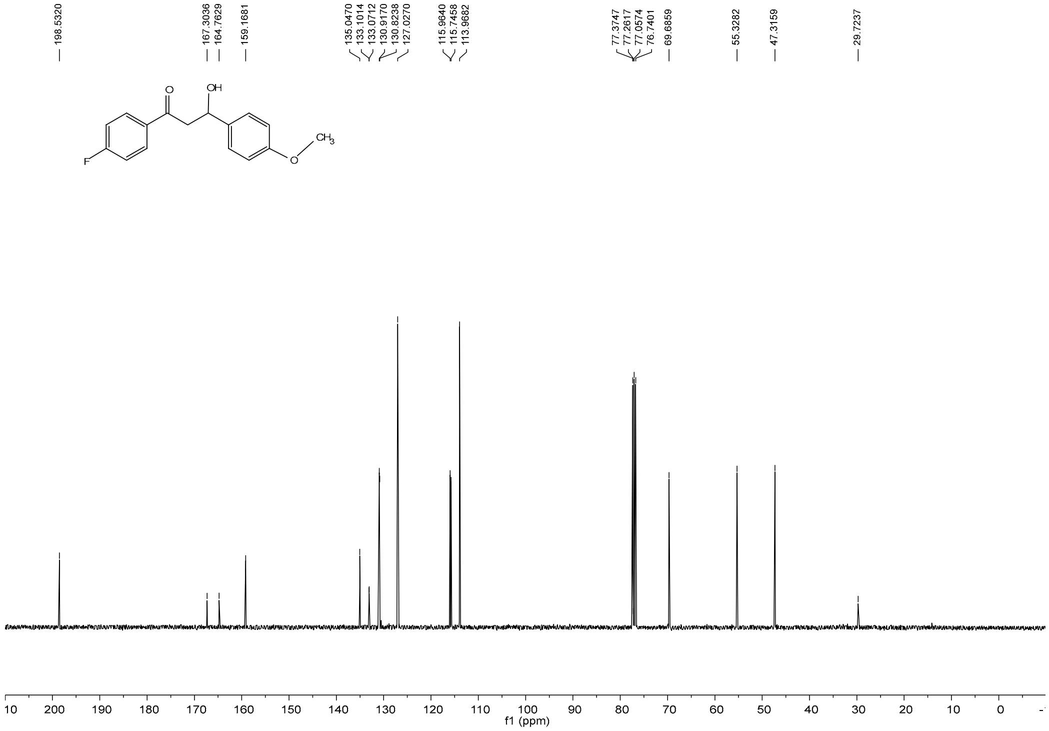
198.5320
167.3036
164.7629
159.1681
135.0470
133.1014
133.0712
130.9170
130.8238
127.0270
115.9640
115.7458
113.9682
77.3747
77.2617
77.0574
76.7401
69.6859
55.3282
47.3159
29.7237
OH
O
F
O
CH3
f1 (ppm)

6r

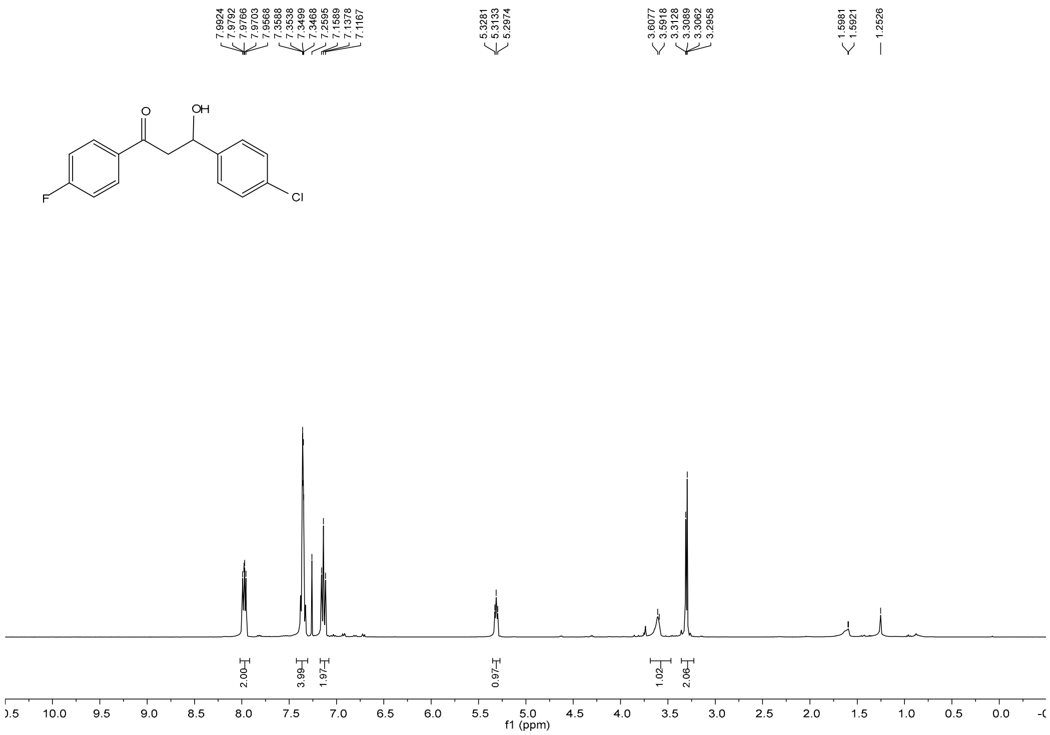
7.9924
7.9792
7.9766
7.9703
7.9568
7.3588
7.3538
7.3499
7.3468
7.2595
7.1589
7.1378
7.1167
5.3281
5.3133
5.2974
3.6077
3.5918
3.3128
3.3089
3.3062
3.2958
1.5981
1.5921
1.2526
O
OH
F
Cl
2.00
3.99
1.97
0.97
1.02
2.06
f1 (ppm)

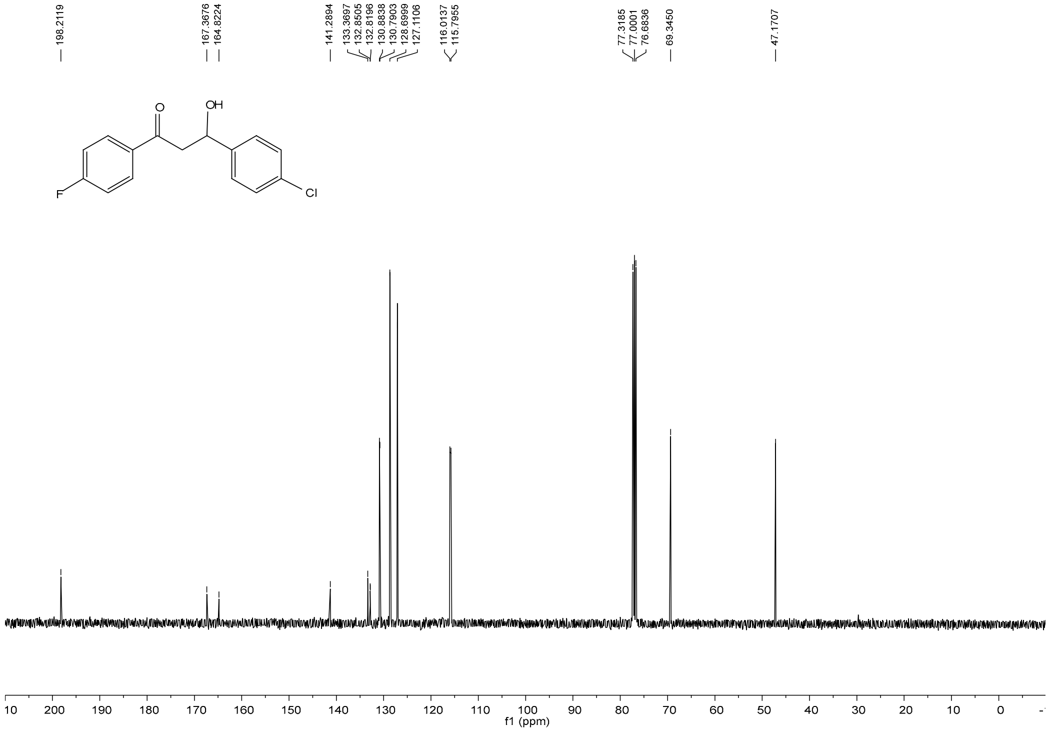
198.2119
167.3676
164.8224
141.2894
133.3697
132.8505
132.8196
130.8838
130.7903
128.6999
127.1106
116.0137
115.7955
77.3185
77.0001
76.6836
69.3450
47.1707
O
OH
F
Cl
f1 (ppm)

6s

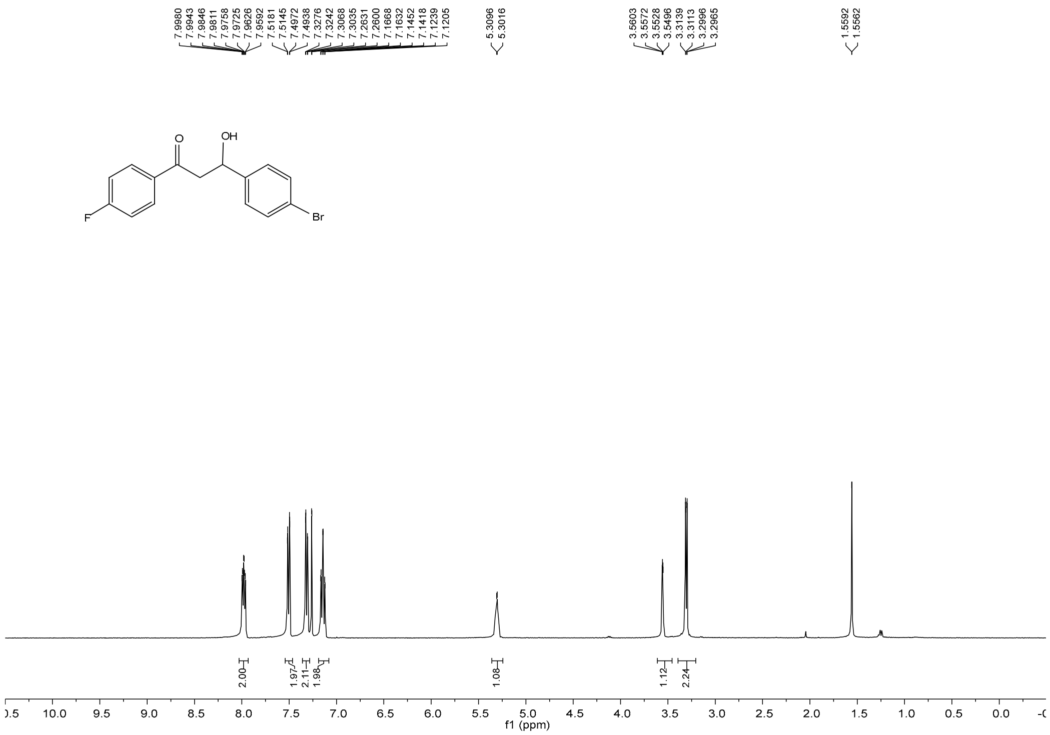
7.9980
7.9943
7.9846
7.9811
7.9758
7.9725
7.9626
7.9592
7.5181
7.5145
7.4972
7.4938
7.3276
7.3242
7.3068
7.3035
7.2631
7.2600
7.1668
7.1632
7.1452
7.1418
7.1239
7.1205
5.3096
5.3016
3.5603
3.5572
3.5528
3.5496
3.3139
3.3113
3.2996
3.2965
1.5592
1.5562
O
OH
F
Br
2.00
1.97
2.11
1.98
1.08
1.12
2.24
f1 (ppm)

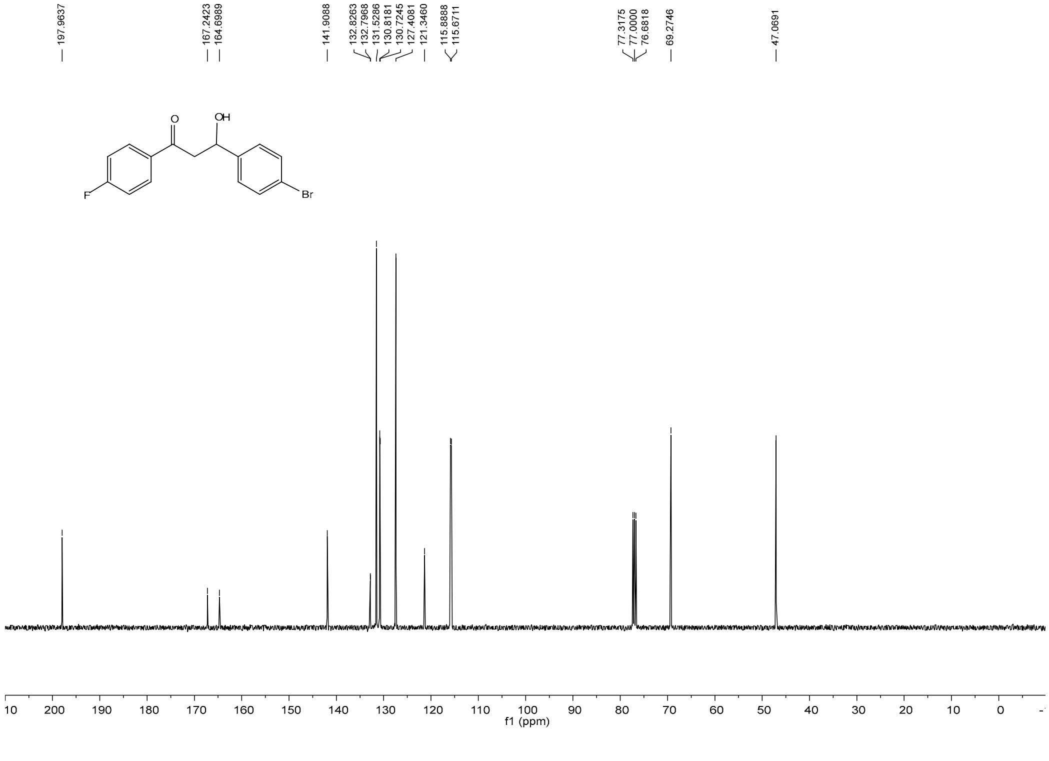
197.9637
167.2423
164.6989
141.9088
132.8263
132.7968
131.5286
130.8181
130.7245
127.4081
121.3460
115.8888
115.6711
77.3175
77.0000
76.6818
69.2746
47.0691
O
OH
F
Br
f1 (ppm)

6t

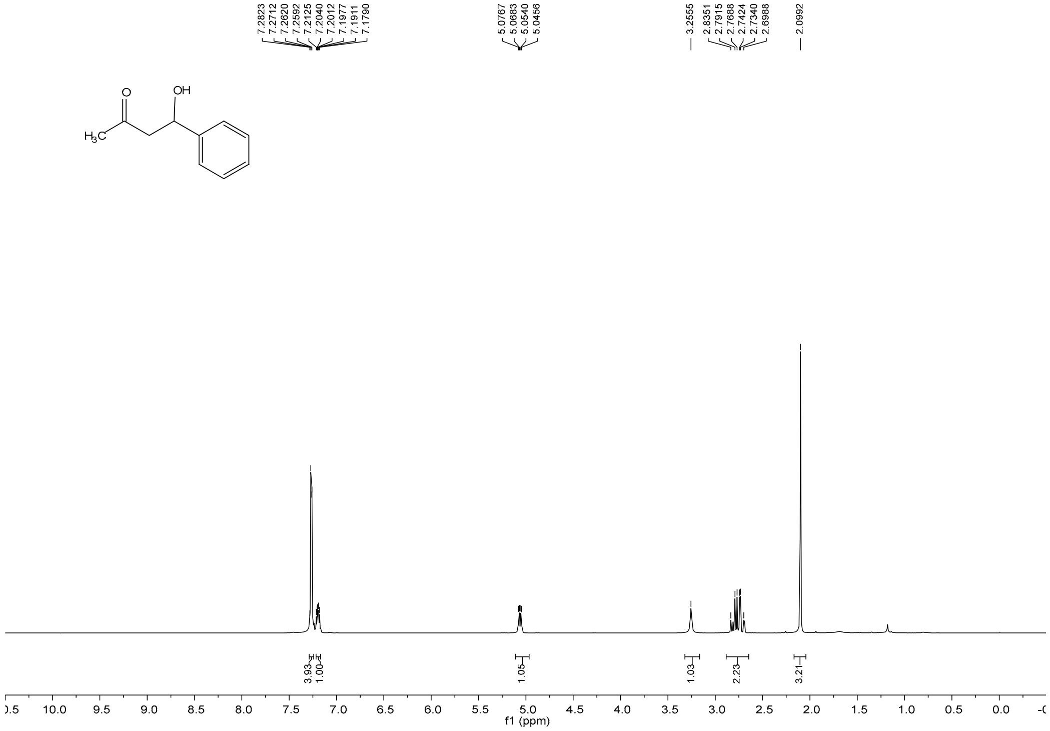
7.2823
7.2712
7.2620
7.2592
7.2125
7.2040
7.2012
7.1977
7.1911
7.1790
5.0767
5.0683
5.0540
5.0456
3.2555
2.8351
2.7915
2.7688
2.7424
2.7340
2.6988
2.0992
OH
O
H3C
3.93
1.00
1.05
1.03
2.23
3.21
f1 (ppm)

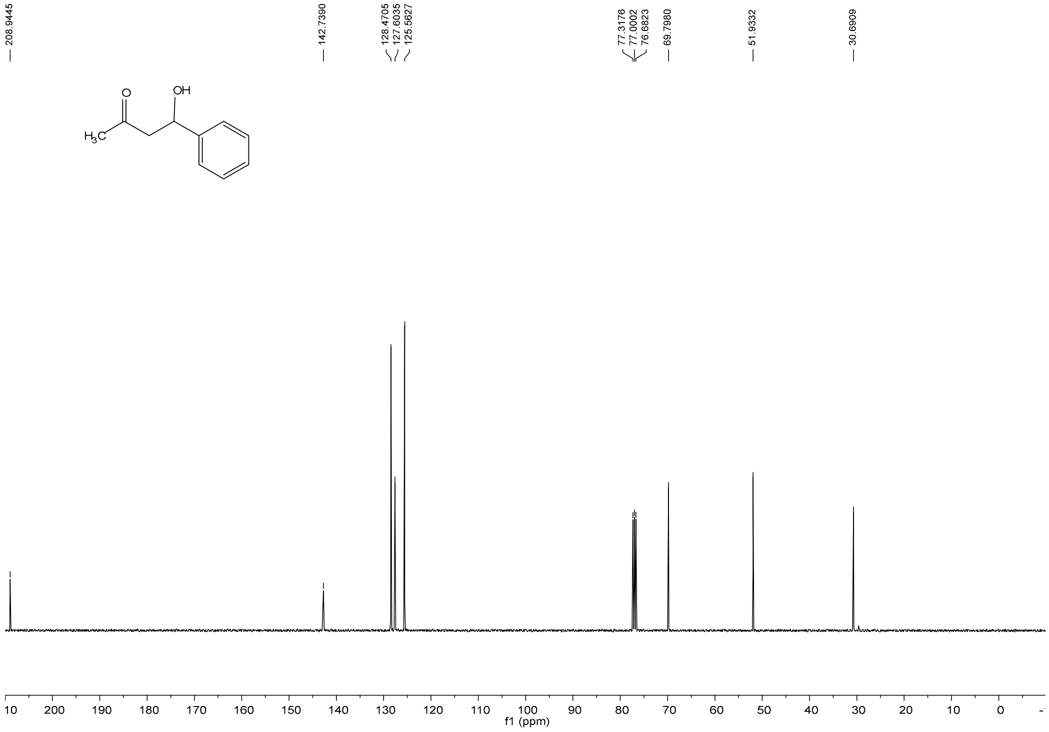
208.9445
142.7390
128.4705
127.6035
125.5627
77.3176
77.0002
76.6823
69.7980
51.9332
30.6909
OH
O
H3C
f1 (ppm)

6u

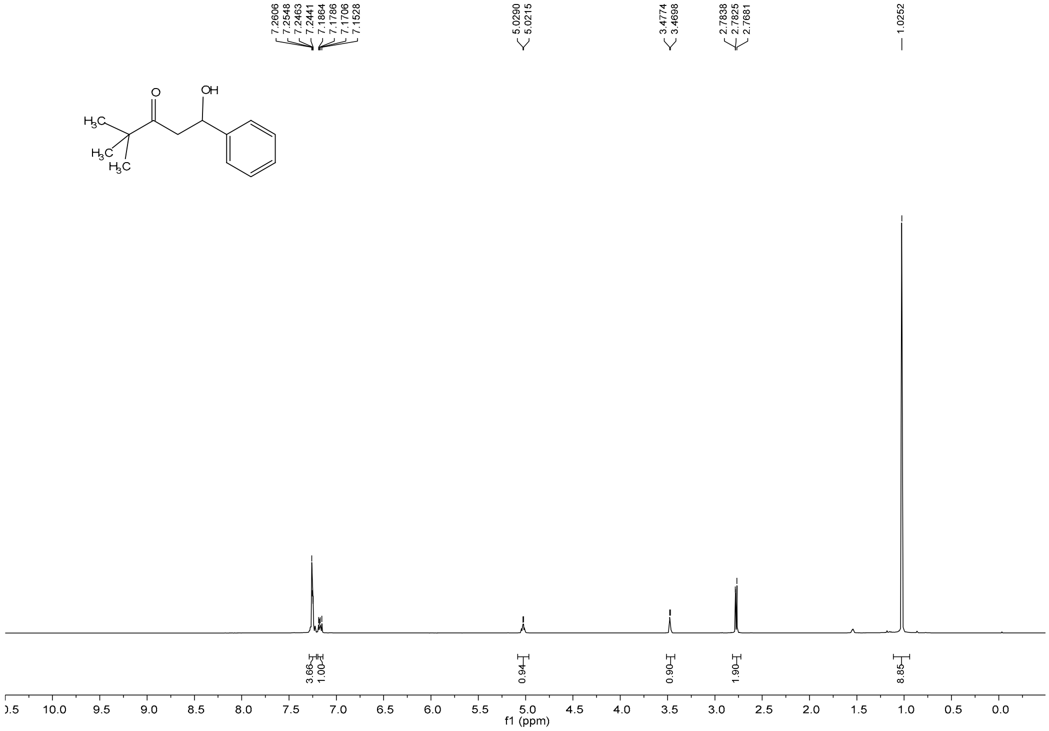
7.2606
7.2548
7.2463
7.2441
7.1864
7.1786
7.1706
7.1528
5.0290
5.0215
3.4774
3.4698
2.7838
2.7825
2.7681
1.0252
O
OH
H3C
H3C
H3C
3.66
1.00
0.94
0.90
1.90
8.85
10.0
9.5
9.0
8.5
8.0
7.5
7.0
6.5
6.0
5.5
5.0
4.5
4.0
3.5
3.0
2.5
2.0
1.5
1.0
0.5
0.0
f1 (ppm)

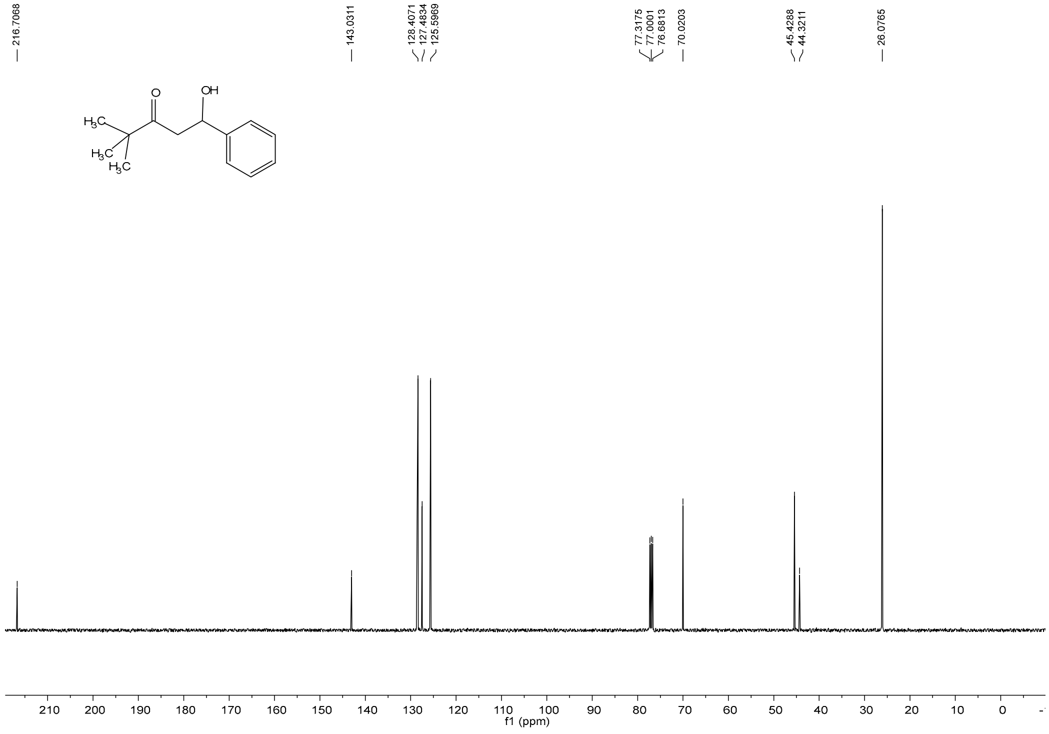
216.7068
143.0311
128.4071
127.4834
125.5969
77.3175
77.0001
76.6813
70.0203
45.4288
44.3211
26.0765
O
OH
H3C
H3C
H3C
210
200
190
180
170
160
150
140
130
120
110
100
90
80
70
60
50
40
30
20
10
0
f1 (ppm)

6v.

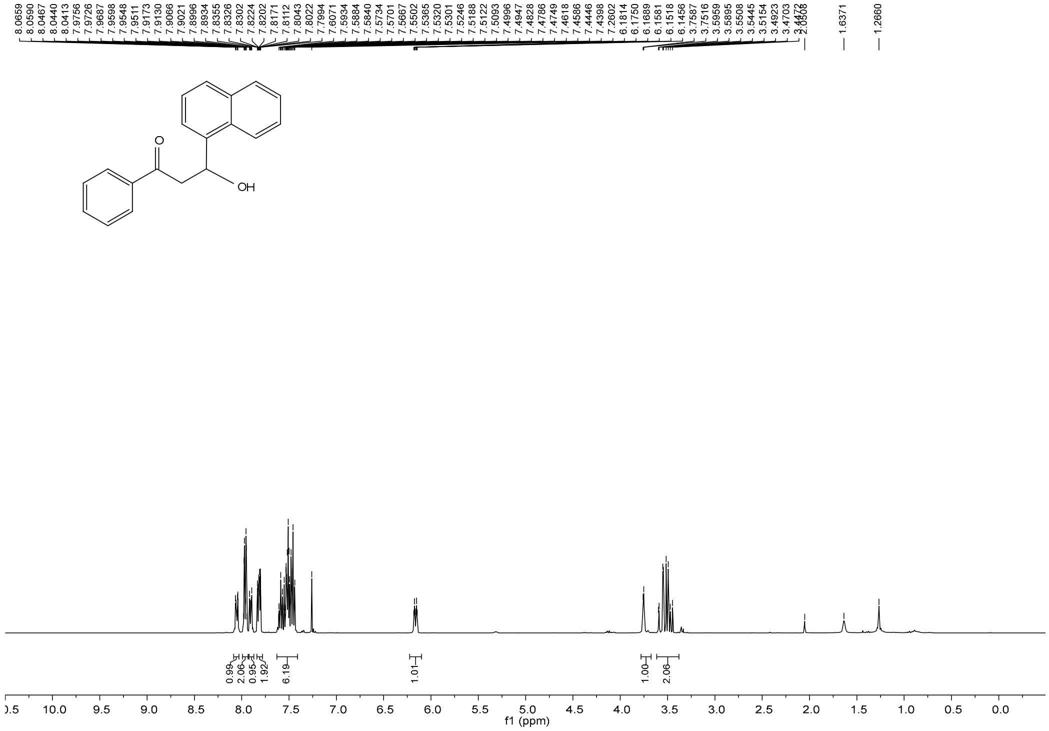

O
OH
f1 (ppm)

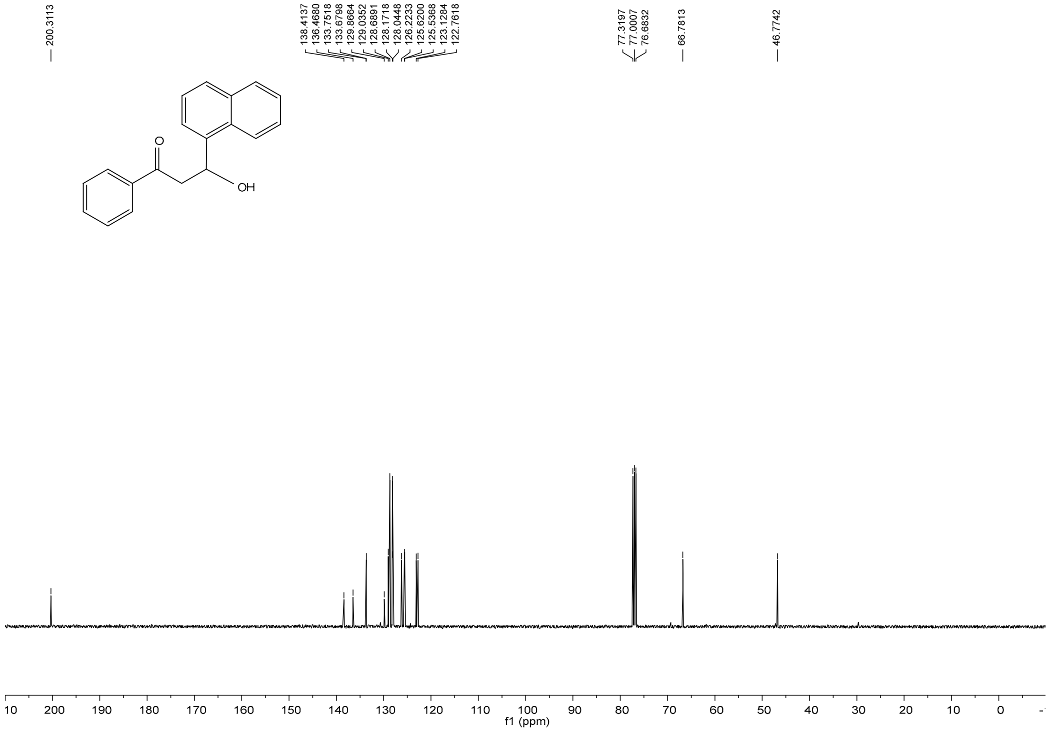

200.3113
138.4137
136.4880
133.7518
133.6798
129.8664
129.0352
128.6891
128.1718
128.0448
126.2233
125.6200
125.5368
123.1284
122.7618
77.3197
77.0007
76.6832
66.7813
46.7742
O
OH
f1 (ppm)

6w

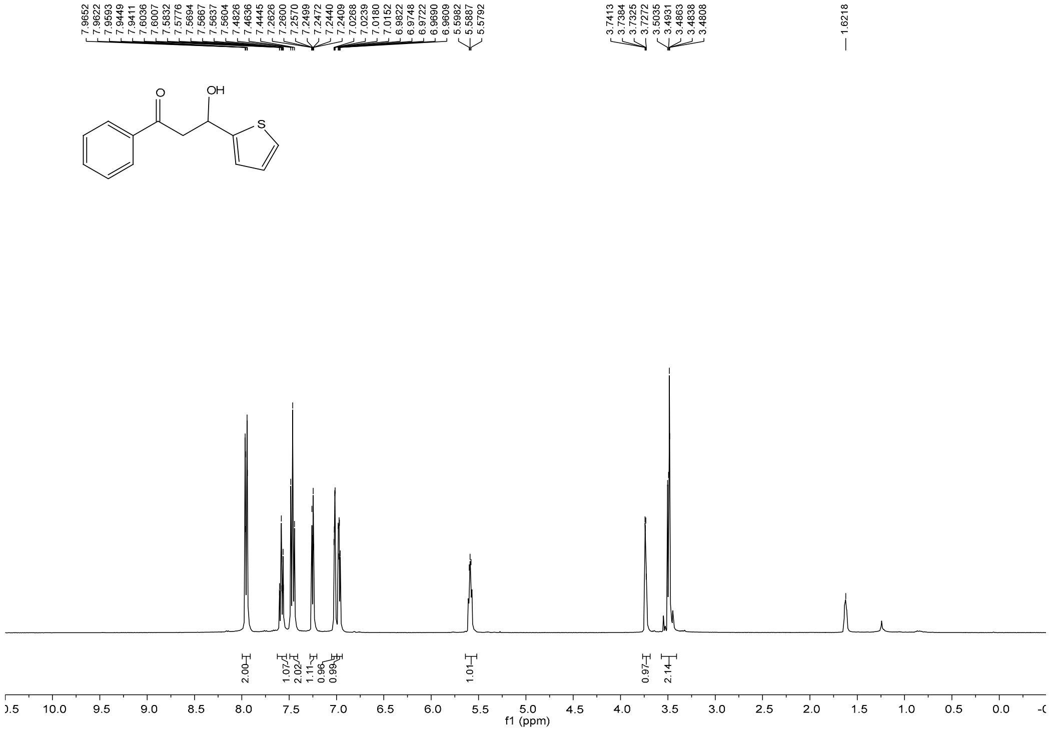
7.9652 7.9622 7.9593 7.9449 7.9411 7.6036 7.6007 7.5832 7.5776 7.5694 7.5667 7.5637 7.5604 7.4826 7.4636 7.4445 7.2626 7.2600 7.2570 7.2499 7.2472 7.2440 7.2409 7.0268 7.0239 7.0180 7.0152 6.9922 6.9748 6.9722 6.9690 6.9609 5.5982 5.5887 5.5792
3.7413 3.7384 3.7325 3.7272 3.5036 3.4931 3.4863 3.4838 3.4808
1.6218
O
OH
S
2.00
1.07
2.02
1.11
0.96
0.99
1.01
0.97
2.14
f1 (ppm)

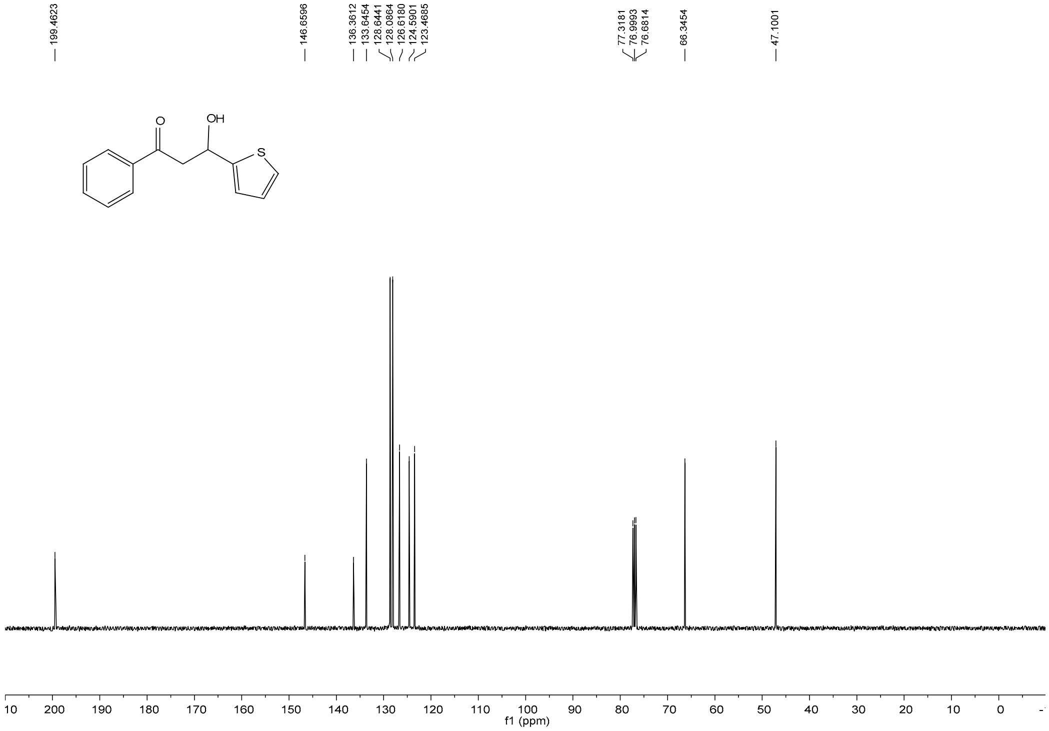
199.4623
146.6596
136.3612 133.6454 128.6441 128.0864 126.6180 124.5901 123.4685
77.3181 76.9993 76.6814
66.3454
47.1001
O
OH
S
f1 (ppm)

6x

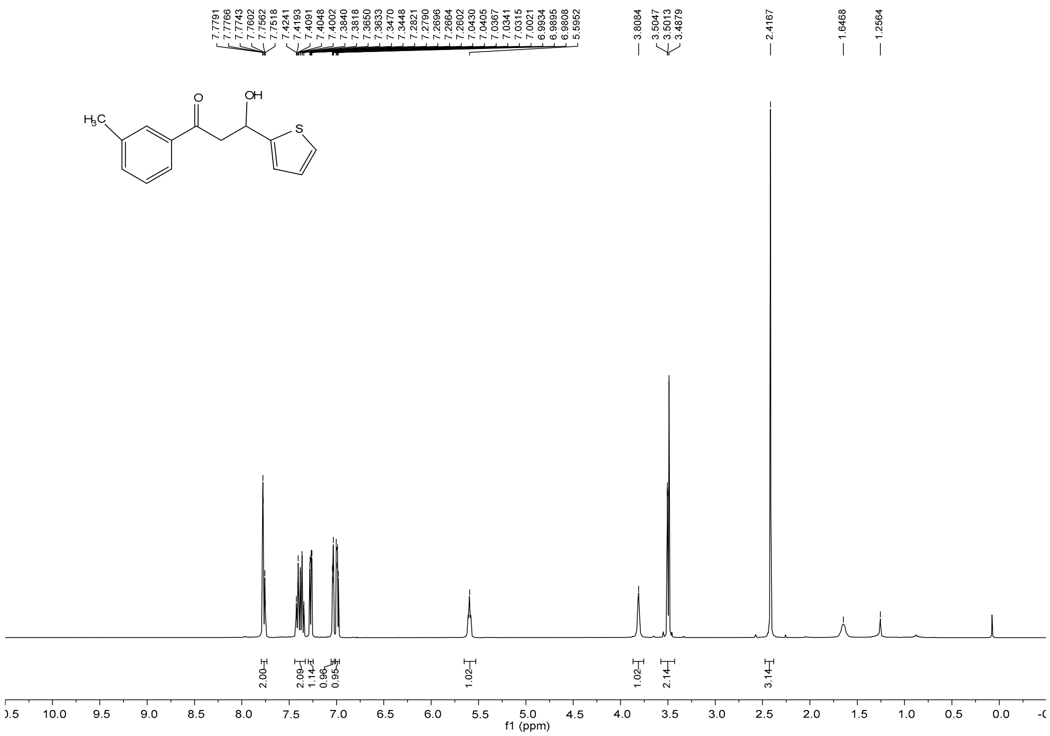

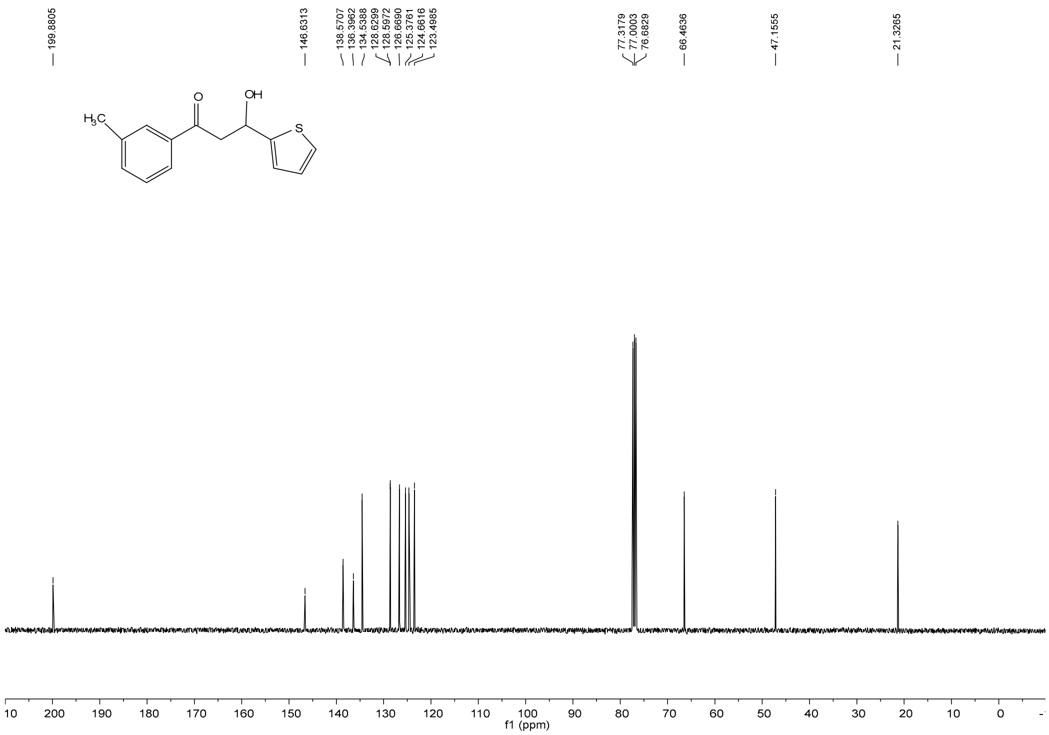

6y

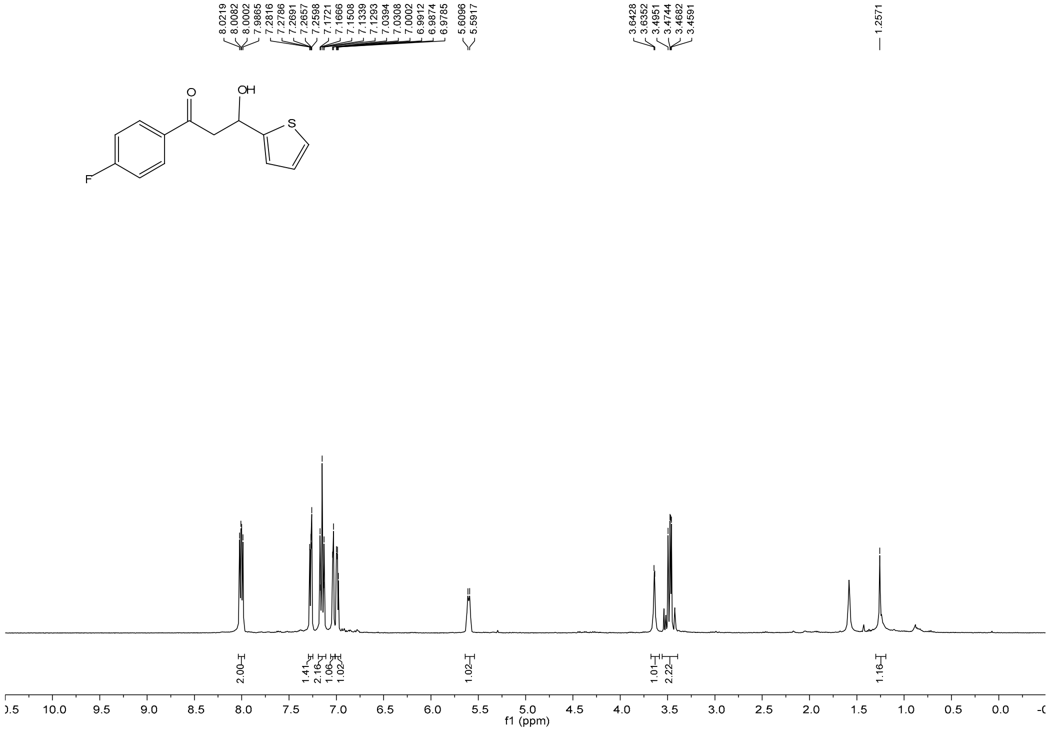
8.0219
8.0082
8.0002
7.9865
7.2816
7.2786
7.2691
7.2657
7.2598
7.1721
7.1666
7.1508
7.1339
7.1293
7.0394
7.0308
7.0002
6.9912
6.9874
6.9785
5.6096
5.5917
3.6428
3.6352
3.4951
3.4744
3.4682
3.4591
1.2571
O
OH
S
F
2.00
1.41
2.16
1.06
1.02
1.02
1.01
2.22
1.16
f1 (ppm)

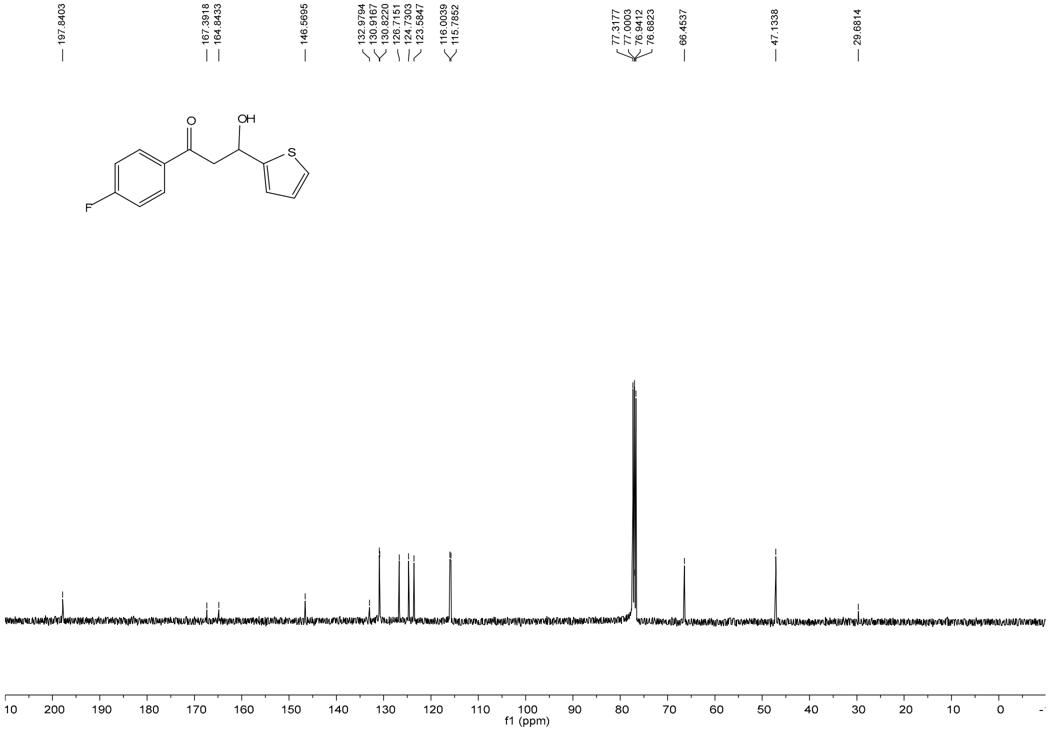
197.8403
167.3918
164.8433
146.5695
132.9794
130.9167
130.8220
126.7151
124.7303
123.5847
116.0039
115.7852
77.3177
77.0003
76.9412
76.6823
66.4537
47.1338
29.6814
O
OH
S
F
f1 (ppm)

6z

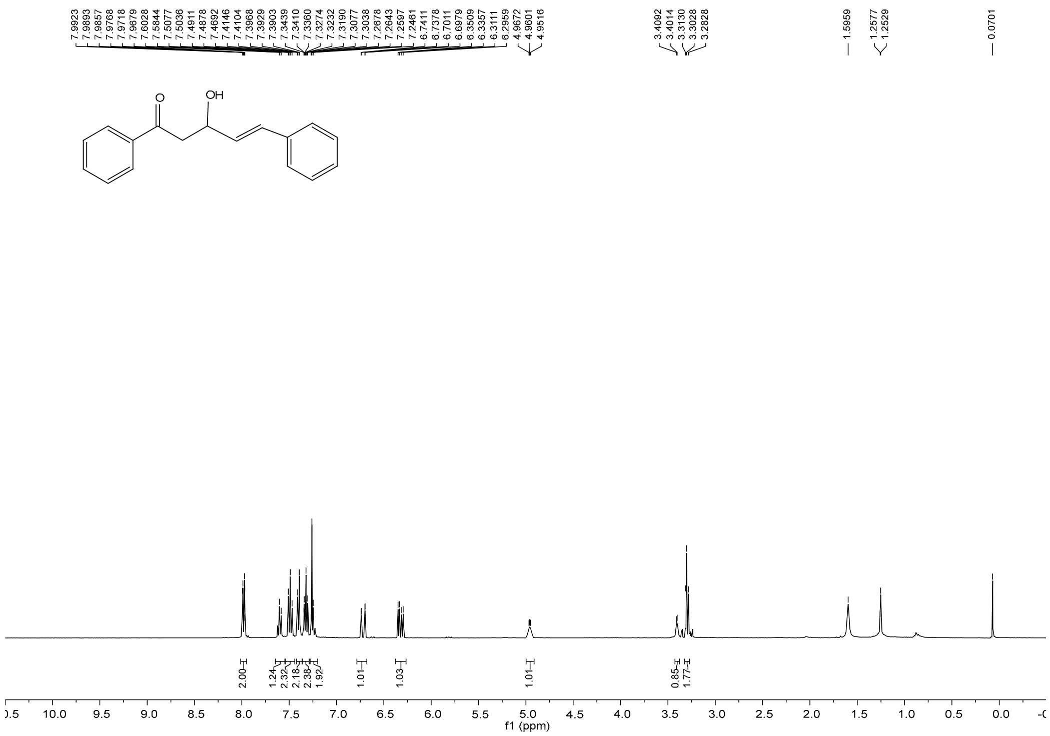

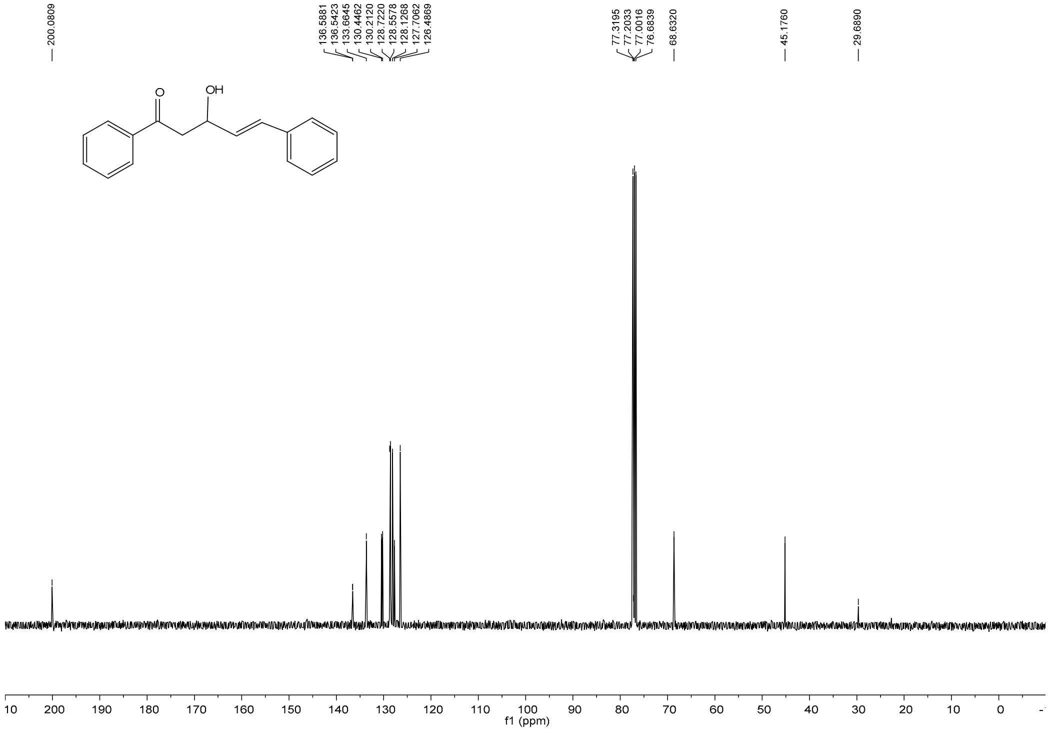

Supplement: Supplementary file 1 [file polymers-11-01417-s001.pdf]
